# Common and separable neural alterations in substance use disorders: A coordinate‐based meta‐analyses of functional neuroimaging studies in humans

**DOI:** 10.1002/hbm.25085

**Published:** 2020-09-10

**Authors:** Benjamin Klugah‐Brown, Xin Di, Jana Zweerings, Klaus Mathiak, Benjamin Becker, Bharat Biswal

**Affiliations:** ^1^ The Clinical Hospital of Chengdu Brain Science Institute, MOE Key Laboratory for Neuroinformation, Center for Information in Medicine, School of Life Science and Technology University of Electronic Science and Technology of China Chengdu Sichuan China; ^2^ Department of Biomedical Engineering New Jersey Institute of Technology Newark New Jersey USA; ^3^ Department of Psychiatry, Psychotherapy and Psychosomatics, Faculty of Medicine RWTH Aachen Aachen Germany; ^4^ JARA Translational Brain Medicine RWTH Aachen Aachen Germany

**Keywords:** alcohol, cannabis, cocaine, cognition, striatum, fMRI, nicotine, reward, substance use disorder

## Abstract

Delineating common and separable neural alterations in substance use disorders (SUD) is imperative to understand the neurobiological basis of the addictive process and to inform substance‐specific treatment strategies. Given numerous functional MRI (fMRI) studies in different SUDs, a meta‐analysis could provide an opportunity to determine robust shared and substance‐specific alterations. The present study employed a coordinate‐based meta‐analysis covering fMRI studies in individuals with addictive cocaine, cannabis, alcohol, and nicotine use. The primary meta‐analysis demonstrated common alterations in primary dorsal striatal, and frontal circuits engaged in reward/salience processing, habit formation, and executive control across different substances and task‐paradigms. Subsequent sub‐analyses revealed substance‐specific alterations in frontal and limbic regions, with marked frontal and insula‐thalamic alterations in alcohol and nicotine use disorders respectively. Examining task‐specific alterations across substances revealed pronounced frontal alterations during cognitive processes yet stronger striatal alterations during reward‐related processes. Finally, an exploratory meta‐analysis revealed that neurofunctional alterations in striatal and frontal reward processing regions can already be determined with a high probability in studies with subjects with comparably short durations of use. Together the findings emphasize the role of dysregulations in frontostriatal circuits and dissociable contributions of these systems in the domains of reward‐related and cognitive processes which may contribute to substance‐specific behavioral alterations.

## Graphical abstract

The present results emphasize the role of dysregulations in striato‐frontal circuits as shared pathological substrate across substance use disorders. In addition, the meta‐analytic findings revealed for the first time evidence for robust substance‐specific alterations in limbic‐thalamo‐frontal circuits that may contribute to substance‐specific behavioral dysregulations and furthermore suggest that dysregulations in the domains of motivational and cognitive processes are mediated by separable brain systems.

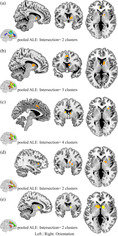

## 1 INTRODUCTION

Problematic use of illicit and licit drugs and substance use disorders represent a major challenge for society, in terms of individual suffering and socioeconomic costs (Degenhardt et al., 2013; Liao, Deng, & Kang, 2010; Rehm & Shield, 2019). Substance use disorders are estimated to contribute to 20% of the world mental illness (Whiteford et al., 2013) and recent large scale surveys estimate that worldwide over 35 million people fulfill the criteria for a substance use disorder (America & America, 2019). Disorders related to alcohol, nicotine, stimulant (e.g., cocaine), and cannabis use are among the most prevalent. Despite increasing treatment demand for problematic use of these substances (European Monitoring Centre for Drugs and Drugs, 2019) treatment options remain limited and of moderate efficacy (van den Brink, 2012).

Based on animal models and human neuroimaging research substance use disorders, particularly addiction as a common pathological endpoint, has been reconceptualized as a chronic relapsing disorder of the brain that is characterized by a preoccupation with drug‐seeking and taking, compulsive use, loss of behavioral control, and withdrawal (DSM‐5) (American Psychiatric Association, 2013). On the neural level, the transition from volitional use to problematic and ultimately compulsive use is driven by progressive dysregulations in the brain's motivational and cognitive circuits, particularly the frontostriatal circuits engaged in incentive salience and reward processing, habit formation, and executive control (Everitt & Robbins, 2016; Koob & Volkow, 2016; Zilverstand, Huang, Alia‐Klein, & Goldstein, 2018).

Based on early animal studies demonstrating that the acute reinforcing effects of all drugs of potential abuse increase dopamine in the terminal regions of the mesocortical‐striatal system including the ventral striatum (Di Chiara & Imperato, 1988)—which with repeated use may drive dysregulations in incentive salience and habit formation (Everitt & Robbins, 2016; Robinson & Berridge, 2001)—most research emphasizes the common neuropathological endpoints across substances and substance use disorders. In line with animal models demonstrating that neuroplastic changes in the striatum mediate exaggerated salience to drug cues at the expense of natural rewards and habitual responses to cues repeatedly paired with the drug (Robbins, Ersche, & Everitt, 2008), exaggerated striatal drug cue reactivity and blunted striatal processing of nondrug rewards has been demonstrated in functional MRI studies in human drug users with regular and addictive use of different substances (Chase, Eickhoff, Laird, & Hogarth, 2011; Kühn & Gallinat, 2011; Vollstädt‐Klein et al., 2010; Zhou et al., 2019; Zimmermann et al., 2019). However, despite convergent evidence for striatal maladaptations across different substance use disorders, substance‐specific predisposing factors (Becker et al., 2015; Cheng et al., 2019; Elsayed et al., 2018; Zilberman, Yadid, Efrati, & Rassovsky, 2019) and addiction‐related alterations have been increasingly recognized, such that frontal regions have been found to be differentially impacted by stimulant or opioid use (Badiani, Belin, Epstein, Calu, & Shaham, 2011) and neurocognitive deficits in domains associated with frontostriatal circuits such as inhibitory control and cognitive flexibility have been found to be differentially impacted by alcohol, stimulants, and cannabis (Fernández‐Serrano, Pérez‐García, & Verdejo‐García, 2011; Smith, Mattick, Jamadar, & Iredale, 2014). Further evidence for substance use disorder‐specific brain alterations comes from a recent qualitative review suggesting that different addictions may be associated with alterations in distinct brain systems and particularly alterations in frontal regions appear to be substance‐specific (Zilberman, Lavidor, Yadid, & Rassovsky, 2019).

The differences might result from common versus substance‐specific predisposing factors that render individuals vulnerable to develop escalation of use in general versus for a particular substance (George & Koob, 2010). Furthermore, differences in the neurobiological effects of the substances may arise from the specific neurotoxic profiles and neurotransmitter systems. In addition to shared effects on the dopamine system, the substances engage different primary neurotransmitter systems (Nestler, 2005), which may lead to transmitter system‐specific neuroadaptations in long‐term users. In addition, the acute rewarding effects of all substances engage the dopamine system leading to the down‐regulation of dopamine receptors (Koob & Volkow, 2016) for chronic users. The acute effects of cannabis are mediated by the endocannabinoid system and regional‐specific downregulation of the cannabinoid CB1 receptor (Hirvonen et al., 2012), the acute effects of nicotine are primarily mediated by its stimulatory effects on neuronal nicotinic acetylcholine receptors (nAChRs) and long‐term nicotine exposure leads to neuroplastic adaptations in nACh receptor expression (Becker & Hurlemann, 2016; Perez, Bordia, McIntosh, Grady, & Quik, 2008), and the acute effects of cocaine are primarily mediated by effects on the dopamine system and marked neuroplastic changes of striatal dopamine receptors have been consistently reported in cocaine use disorder (Payer et al., 2014; Schlaepfer, Pearlson, Wong, Marenco, & Dannals, 1997).

Despite emerging evidence for common but also substance‐specific neurobiological alterations, most previous research emphasized common pathological pathways. Although the determination of common pathways of addiction may promote the development of general treatment approaches, the identification of substance‐specific neurobiological mechanisms is essential to further enhance our understanding of predisposing factors as well as to develop specialized treatment options. To address common limitations of single studies such as low sample size, study‐specific characteristics of the sample and inclusion of one substance only, the present study employed a meta‐analytic approach covering previous task fMRI studies on alcohol, cannabis, cocaine, and nicotine substance use disorder to determine common and disorder‐specific neural alterations. To this end, we conducted a quantitative coordinate‐based meta‐analysis (CBMA) covering previous fMRI studies in substance use populations employing whole‐brain foci from the studies selected according to our inclusion criteria. The CBMA approach was preferred to other methods like image‐based meta‐analysis because it takes advantage of the published coordinates, and quantitatively provides a summary of the presented results under the specific research question, while the latter approach is limited by the availability of whole‐brain images (statistical images are currently only availiable for a limited number of studies). We first conducted a main ALE analysis to determine core regions that neurally underpin substance use disorders across substances. This was followed by sub‐meta‐analyses employing substance‐specific subtraction and conjunction analysis to further specify common and substance‐specific neural alterations as well as functional domain‐specific alterations for reward and cognitive processes. Based on previous animal models and human imaging research, we hypothesized common alterations in striatal systems engaged in reward/motivation (ventral striatum) and habit formation (dorsal striatum) as well as partly dissociable effects on frontal systems engaged in executive control and behavioral regulation. Moreover, we examined whether the observed substance‐specific alterations are driven by an interaction between the substance used and the class of task paradigms employed.

## 2 METHODS

### 2.1 Literature selection

**FIGURE 1:**
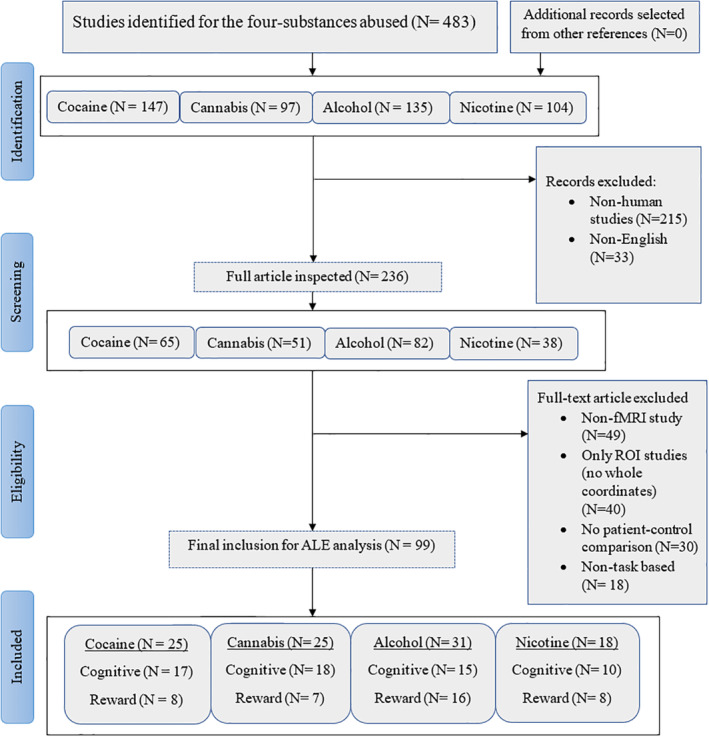
PRISMA procedure for inclusion of articles

We obtained articles including four kinds of substances that are regularly abused namely cocaine, cannabis, alcohol, and nicotine (cigarettes or tobacco). Utilizing Scopus, PubMed, and Web of Science, peer‐reviewed studies published between January 1, 2000 and November 1, 2019 were collected using the following search terms; “Alcohol” or “Cocaine” or “Cannabis” or “Nicotine/Tobacco/cigarette” and “Functional magnetic resonance imaging” or “fMRI.” The reference list of the selected articles was inspected separately. We targeted articles that reported: whole‐brain coordinates either in the main paper or supplementary material with stereoscope coordinates in either Talairach or MNI (Montreal Neurological Institute) space, comparisons between healthy controls and patients with substance dependency or heavy usage. The exclusion criteria were as follows: (a) Articles reporting only region‐of‐interest (ROI) results (if the study additionally reported whole‐brain corrected findings these were included), (b) Articles with poly‐drug users and high comorbidities with psychiatric or somatic disorders (e.g., schizophrenia or HIV), (c) Articles focusing on parental exposure, and (d) Articles reporting results from the exact same data set from previous studies. The breakdown of article screening and exclusion for the main and sub‐meta‐analysis is shown in Figure 1.

### 2.2 Study approach: Activation likelihood estimation

In initial meta‐analyses, the brain functional alterations for each substance use disorders (SUD) were computed relative to their respective control groups. Next, we used a three‐step approach (a) to establish brain functional alterations across all SUDs in comparison to their control groups, (b) to establish common and distinguishable alterations between the SUDs by employing conjunction and differential contrasts that compare the SUD‐specific activation likelihood estimation (ALE) maps (SUD vs. control groups, separately computed for each SUD category and their respective control groups) between the SUD categories by means of conjunction and subtraction analysis, and, (c) to investigate whether common neurofunctional alterations across the SUDs are related to the classes of experimental task paradigms employed or by the effects of the drug use in the study populations.

In line with the study goals, we performed a series of coordinate‐based meta‐analyses using the GingerALE 3.0.2 command‐line version (Eickhoff et al., 2009; Eickhoff et al., 2011; Eickhoff, Bzdok, Laird, Kurth, & Fox, 2012), specifically to compute (a) individual ALE in each SUD, (b) subtraction and conjunction ALEs between each pair of SUD category. In each step of the analysis, all foci in MNI space were converted to Talairach space. To address the spatial uncertainty linked to the foci, the three‐dimensional Gaussian probability distribution was applied to centers of the coordinates and images derived from the foci for all experiments obtained as modeled activation (MA). It is worth noting that ALE tries to find a strong agreement between the included experiments. This is achieved by computing the union between the generated maps while taking into account the disparities between true activation and noise. For all meta‐analyses family‐wise error correction (FWE) with a corrected *p* < .05 on the cluster‐level was applied to control for multiple comparisons. In line with recent recommendations for the application of cluster‐based correction methods (Eickhoff et al., 2016) an initial cluster forming threshold of *p* < .001 was employed.

#### 2.2.1 Step 1: Direct comparison of ALEs in individual SUD categories

We computed single group ALE analysis for the four substances, using the parameters described above. Coordinates from each substance group were combined as one study before computing the ALE. This was done to ascertain the overall maximum activations across the SUD.

#### 2.2.2 Step 2: Subtraction and conjunction analysis between pairs of SUD categories

We examined differences and overlaps between all six pairs of substances. The comparisons were done without specific a priori hypotheses (although co‐use of the substances is often reported in research, (see [Karriker‐Jaffe, Subbaraman, Greenfield, & Kerr, 2018]) in the following manner: cannabis versus cocaine, alcohol versus cocaine, alcohol versus cannabis, cannabis versus nicotine, cocaine versus nicotine, and nicotine versus alcohol. ALE analysis for individual substance groups was conducted first. The pooled foci were further used to compute the cluster‐level FWE corrected maps and subsequently to obtain ALE images. Furthermore, we conducted a subtraction analysis to obtain the differences in ALE between the substance groups. In all of the results, clusters of brain regions were identified including the number of studies that contributed to them.

#### 2.2.3 Step 3: Post hoc analysis based on the functional domain

Based on the results in Step 2, we divided pairs of SUD categories that had conjunction (matched/overlapped pairs) activation into two categories of task paradigms that reflected distinct functional domains; that is, “Reward” comprising the processing of reward, motivation, or anticipation and “Cognition” with cognitive‐control, behavior, and emotion tasks. Despite conceptual frameworks proposing differential alterations with respect to the processing of drug‐associated rewards (particularly drug cue reactivity) and natural rewards, specifically exaggerated reactivity to drug‐associated rewards and attenuated reactivity to nondrug rewards we decided to pool these studies under the domain reward processing. This decision increased the power of the corresponding meta‐analyses and additionally adhered to the main aim of the study which was to determine neurofunctional alterations in SUD while not further disentangling hyper‐ from hypo‐activations on the analytic level. The examination of the different paradigm domains serves the primary purpose to investigate whether the conjunctions were influenced by the experimental design or solely by the substances abused. To this end we computed whole‐brain ALE firstly for all studies based on the experimental task, secondly, we grouped the foci into patient and control per each SUD category and computed the cluster ALE to ascertain the group interactions under the two categories of experimental paradigms. We also extracted the foci generated by the probabilities of functional change from each cluster including the task associated, using the Kruskal‐Willies test we computed the dependency test to ensure homogeneity between the two categories of tasks forming the conjunction. Significant threshold was set to *p* < .05. Finally, to explore we conducted an exploratory meta‐regression based on the duration of drug used in years and the MA values obtained from the combined meta‐analysis describing the common neurofunctional alterations across all SUD using spearman rank correlation with 95% confidence level. The main purpose of this analysis was to explore whether the identified regions differentially vary in their ALE probability with respect to the duration of drug use.

## 3 RESULTS

### 3.1 Included study sample characteristics

Out of the 99 studies included in the meta‐analysis, cocaine studies contributed to 30% (828) of the total foci, while cannabis, alcohol, and nicotine contributed to 23.01% (637), 27.45% (760), and 19.44% (538), respectively of the total foci analyzed, indicating no bias toward a single substance for the ALE computations. Table 1 shows the demography of the four groups of substance abuse study categories and the type of experimental task used in each SUD study. The combined data set yielded data from a total of 2,692 substance users (mean (*SD*) age, 33.9 (11.7)) and 2,564 control subjects (mean (*SD*) age, 31.3 (11.6)), with no significant difference between the four SUD groups *χ*^2^ = 1.3, *p* = 0.7.

**TABLE 1:** Subject characteristics for each study in a group

Study source	Participants (N)		Age, mean (*SD*)		Type of experiment/task
	SUD	HC	SUD	HC	
Cocaine studies					
(Barrós‐Loscertales et al., 2011)	16	16	34.38(7.15)	34.2(8.86)	Stroop task
(Barrós‐Loscertales et al., in press)	30	28	35.9(6.31)	38.89(10.5)	Stop‐signal task
(Bustamante et al., 2011)	15	15	32.4(7.56)	34.2(8.86)	Verbal working memory task
(Caldwell et al., 2015)	219	87	34.9(8.08)	32.15(9.07)	Moral judgment task
(Crunelle et al., 2015)	51	32	32(8)	33(9)	Emotional face matching task
(Ersche et al., 2013)	18	18	34.3(7.2)	32.7(6.9)	Stroop task
(Garavan et al., 2000)	31	17	34(0.5)	26(0.7)	Working memory task
(Ide, Hu, Zhang, Mujica‐Parodi, & Li, 2016)	75	88	39.9(7.6)	38.7(10.9)	Stop signal task
(Kaag et al., 2016)	40	51	31.3(7.9)	31(8.5)	Fear conditioning paradigm
(Kaag, Reneman, Homberg, van den Brink, & van Wingen, 2018)	59	58	31.4(7.6)	30.5(8.1)	Cue reactivity paradigm
(Kirschner et al., 2018)	22	28	29.73(7.99)	28.2(6.72)	Prospective imagery task
(Kober et al., 2016)	30	73	43.78(13.06)	32.22(11.06)	Craving/emotional response task
(Ma et al., 2015)	13	10	37.4(5.3)	35.2(7.3)	Go/NoGo task
(McHugh, Gu, Yang, Adinoff, & Stein, 2017)	45	22	43.42(7.04)	42.05(8.4)	Wisconsin card sorting task
(Mitchell et al., 1998)	15	15	39(10.4)	40.9(7.4)	Stroop task
(Moeller et al., 2010)	19	14	40.8(8.4)	34.5(1.8)	Working memory task
(Moeller et al., 2015)	37	55	43.62(6.7)	40.28(7.44)	Stroop task
(Moeller et al., 2015)	33	20	43.55(8.3)	39.6(5.5)	Inhibitory control task
(Moeller et al., 2018)	37	26	46.05(8.3)	43.1(7.2)	Drug‐choice task
(Potenza, Hong, Lacadie, Fulbright, & Tuit, 2012)	30	36	36.9(6.4)	31.2(9)	Individualized scripts for stress
(Sinha et al., 2005)	20	8	38.75(4.77)	32.8(4.74)	Stress and neutral script
(Tau et al., 2014)	13	13	37.7(6.8)	36.6(6)	Reward‐based spatial learning task
(Worhunsky et al., 2013)	20	20	38.6(9.3)	36.8(8.9)	Stroop task
(Yip et al., 2016)	20	21	38.6(9.29)	34.57(11.99)	Monetary incentive delay task
(Zhang et al., 2016)	100	100	40.3(7.4)	38(10.6)	Stop signal task
Cannabis studies					
(Abdullaev, Posner, Nunnally, & Dishion, 2010)	14	14	19.5(0.8)	19.7(1.4)	Attention network task
(Ames et al., 2013)	16	17	21.15(1.9)	20.27(2.3)	Implicit association test
(Chang, Yakupov, Cloak, & Ernst, 2006)	24	19	28.77(2.81)	30.57(1.83)	Nonverbal visual‐attention task
(Cousijn et al., 2014)	32	41	21.65(2.4)	22.25(2.35)	N‐back task
(De Bellis et al., 2013)	15	41	16.4(7.3)	16(1.2)	Decision‐reward uncertainty task
(Enzi et al., 2015)	15	15	26.33(2.94)	27.13(8.85)	Monetary incentive delay task
(Filbey et al., 2016)	53	68	30.66(7.48)	31.41(10.2)	Cannabis cue‐exposure task
(Filbey, Schacht, Myers, Chavez, & Hutchison, 2009)	38	25	23.74(7.25)	22.04(5.63)	Cue‐elicited craving paradigm
(Gilman et al., 2016)	20	23	20.6(2.5)	21.6(1.9)	Visual discrimination task
(Gruber, Rogowska, & Yurgelun‐Todd, 2009)	15	15	25(8.8)	26(9.0)	Masked affective tasks
(Harding et al., 2012)	21	21	36.5(8.8)	31(11.7)	Multi‐source interference task
(Heitzeg, Cope, Martz, Hardee, & Zucker, 2015)	20	20	19.84(1.45)	20.51(1.26)	Emotion‐arousal word task
(Kanayama, Rogowska, Pope, Gruber, & Yurgelun‐Todd, 2004)	12	10	37.9(7.4)	27.8(7.9)	Working memory task
(Kober, Devito, Deleone, Carroll, & Potenza, 2014)	20	20	26.65(9.81)	29.2(10.06)	Stroop task
(Milivojevic, Constable, & Sinha, 2005)	8	18	36(7.5)	37.2(5.6)	Script‐guided imagery paradigm
(Lopez‐Larson et al., 2012)	24	24	18.2(0.7)	18(1.9)	Finger‐tapping task
(Ma et al., 2018)	23	23	28.2(3.5)	28.7(3.7)	N‐back working memory task
(Nestor, Roberts, Garavan, & Hester, 2008)	49	52	23.35(0.95)	23.05(0.85)	Face‐name task
(Schweinsburg et al., 2008)	15	17	18.1(0.7)	17.9(1.0)	Spatial working memory task
(Tervo‐Clemmens et al., 2018)	22	63	14.12(0.33)	14.21(0.37)	Spatial working memory task
(Tervo‐Clemmens et al., 2018)	14	15	28.16(0.69)	28.16(0.71)	Visuospatial working memory task
(van Hell et al., 2010)	14	13	24.5(4.45)	24(2.7)	Monetary reward task was
(Wesley, Hanlon, & Porrino, 2011)	16	16	26.4(3.6)	26.6(6.1)	Iowa gambling task
(Zimmermann et al., 2019)	23	23	23.86(3.36)	23.67(2.88)	Interpersonal touch paradigm
(Zimmermann et al., 2017)	23	20	21.24(2.59)	21.1(3.61)	Event‐related cognitive reappraisal
Alcohol studies					
(Akine et al., 2007)	9	9	34.6(6.5)	36.2(7.2)	Long‐term memory retrieval task
(Bagga et al., 2014)	18	18	36.5(5.0)	35.2(3.7)	Abstract reasoning task
(Beylergil et al., 2017)	34	26	44.73(8.3)	41.92(9.6)	Reward‐guided decision‐making task
(Brumback et al., 2015)	22	16	17.93(0.7)	17.42(0.7)	Alcohol pictures cue reactivity task
(Chanraud et al., 2009)	24	24	47.8(7.7)	45(5.6)	Free and cued selective reminding test verbal episodic memory assessment
(Dager et al., 2014)	23	33	18.9(0.6)	18.7(0.4)	Figural memory task
(Deserno et al., 2015)	13	14	45.08(6)	43.86(9.2)	Reversal learning task reversal
(Gilman et al., 2015)	18	18	37.7(7.8)	34.5(8.0)	Risk‐taking task
(Gorka, Kreutzer, Petrey, Radoman, & Phan, 2019)	38	27	23.8(3.0)	24.3(2.8)	Startle threat task
(Grodin, Lim, MacKillop, Karno, & Ray, 2019)	24	22	36.41(14)	32.29(9.9)	Cue reactivity task
(Grodin, Steckler, & Momenan, 2016)	17	17	32.25(6.9)	27.72(4.3)	Monetary incentive delay task
(Grüsser et al., 2004)	10	10	36(11.0)	41(8.0)	Cue response task
(Heinz et al., 2007)	12	12	39(7.0)	40(8.0)	Cue response task
(Hermann et al., 2006)	10	10	40(7.0)	38(5.0)	Cue response task
(Hu, Ide, Zhang, Sinha, & Li, 2015)	24	70	38.7(8.3)	35.1(9.9)	Stop signal task
(Jang et al., 2007)	20	20	43.5(6.0)	44.5(7.4)	Mixed cognitive tests
(Jansen et al., 2019)	39	39	41.64(8.6)	44.06(11.0)	Emotion reappraisal task
(Kienast et al., 2013)	11	13	41.9(7.0)	43.2(9.5)	Emotional task
(Maurage, Bestelmeyer, Rouger, Charest, & Belin, 2013)	12	12	24.2(4.5)	23.4(4.2)	Two‐alternative forced choice task
(Park et al., 2007)	9	9	23.22(2.5)	23(2.6)	Cue response task
(Reiter et al., 2016)	43	35	44.42(10.21)	42.00(10.49)	Anticorrelated decision‐making task
(Sjoerds et al., 2013)	31	19	48.5(8.5)	47.7(11.0)	Instrumental learning task was
(Squeglia, Schweinsburg, Pulido, & Tapert, 2011)	40	55	17.9(0.9)	17.88(1.0)	Spatial working memory task
(Wesley, Lile, Fillmore, & Porrino, 2017)	24	11	33.3(8.4)	28.8(7.8)	The N‐Back working memory task
(Wetherill, Castro, Squeglia, & Tapert, 2013)	40	20	18.4(2.1)	18.3(1.4)	Go/no‐go task
(Wiers et al., 2015)	38	17	44.39(7.3)	42.71(9.2)	Cue reactivity task
(Worbe et al., 2014)	19	21	23.21(3.52)	24.14(3.13)	Risk‐taking task
(Wrase et al., 2002)	37	44	43.5(8.7)	34.2(9.3)	Cue reactivity task
(Wrase et al., 2007)	16	16	42.38(7.52)	39.94(8.59)	Monetary incentive delay task
(Yang et al., 2013)	15	15	42.3(7.1)	45.5(8.5)	Anticipatory anxiety paradigm
(Yoon et al., 2009)	12	12	32(5.2)	31(6.2)	Memory encoding tasks
Nicotine studies					
(Artiges et al., 2009)	13	13	26(4.0)	24(4.0)	Smoking cues emotion recognition task
(Carroll, Sutherland, Salmeron, Ross, & Stein, 2015)	23	19	35(10)	30.2(7.2)	Speeded flanker task
(Bühler et al., 2010)	21	21	28(4.3)	25.7(6.1)	Event‐related instrumental motivation task
(Galván et al., 2013)	18	25	19.47(1.33)	19.08(1.15)	Balloon analogue risk task
(Hong et al., 2017)	17	16	39.9(4.9)	39.2(5.2)	Cue reactivity task
(Kobiella et al., 2014)	27	33	41.3(7.9)	41.3(7.9)	Intertemporal choice task
(Okuyemi et al., 2006)	17	17	37.65(9.4)	35.8(10.95)	Cue viewing task
(Lawn et al., in press)	19	19	29.5(10.7)	22.7(4.4)	Value‐based decision‐making task
(Lesage et al., 2017)	24	20	35.8(9.9)	30.4(7.2)	Probabilistic reversal learning task
(Liberman et al., 2018)	5	5	21.7(3.8)	21.7(3.8)	Cue viewing task
(Luijten et al., 2013)	25	23	22.56(2.84)	21.74(1.82)	Go/NoGo task
(Luo, Ainslie, Giragosian, & Monterosso, 2011)	35	36	34.1(7.9)	31.3(7.1)	Adaptive intertemporal choice task
(Maynard, Brooks, Munafò, & Leonards, 2017)	39	19	21.95(3.5)	24(3)	Memory task
(Rose et al., 2013)	28	28	32.68(10.02)	30.11(7.83)	Monetary incentive delay task
(Rubinstein, Luks, Dryden, Rait, & Simpson, 2011)	12	12	16(1.4)	16(1.4)	Cue reactivity paradigm
(Wagner, Cin, Sargent, Kelley, & Heatherton, 2011)	17	17	23.1(NA)	21.4(NA)	Cue response task
(Weywadt, Kiehl, & Claus, 2017)	81	38	59(1.5)	61(1.36)	Go/no‐go task
(Yalachkov, Kaiser, Görres, Seehaus, & Naumer, 2012)	15	15	28.3(3.7)	27(5.01)	Visual stimuli

*Note*: References for the included articles can be found in the supplementary material.

Abbreviations: HC, healthy controls; N, the number of participants; *SD*, standard deviation; SUD, substance use disorder.

### 3.2 Step 1: Combined ALE analysis of all SUDs

**FIGURE 2:**
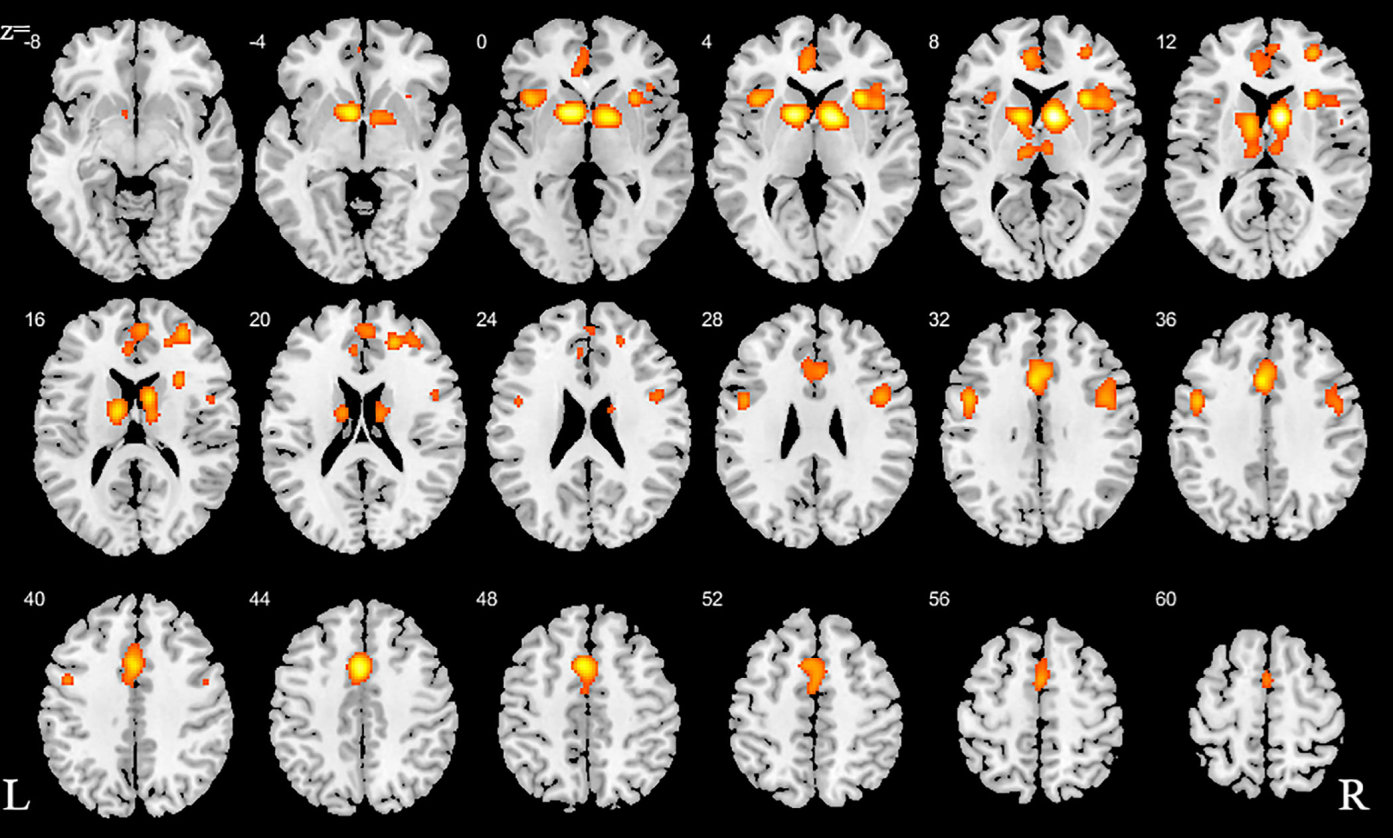
ALE for combined studies. All slices in transverse view with ascending slice number. Displayed at FWE < 0.05

We initially conducted a main meta‐analysis that incorporated the ALE maps from all SUDs versus the respective control groups (Step 1). This analysis aimed at determining neurofunctional alterations across all SUDs and revealed neurofunctional alterations primarily located in the dorsal striatum, including the caudate and putamen as well as the prefrontal, limbic and insular cortex, including inferior, superior, and medial frontal regions as well as the anterior cingulate cortex (ACC) and the anterior insula (Figure 2, Table 2).

**TABLE 2:** Detailed peak coordinates for the combined studies with the number of clusters for each volume

Cluster #	x	y	z	Vol	Label	No. contributing studies
1	10	6	10	15,272	Right cerebrum. Sub‐lobar: Caudate	46
1	−14	8	2		Left cerebrum. Sub‐lobar. Lentiform nucleus: Putamen	
1	−12	−4	16		Left cerebrum. Sub‐lobar: Caudate	
1	12	−6	18		Right cerebrum. Sub‐lobar: Caudate	
1	6	−16	10		Right cerebrum. Sub‐lobar: Thalamus‐dorsal nucleus	
1	−10	−18	8		Left cerebrum. Sub‐lobar: Thalamus‐dorsal nucleus	
2	−2	10	44	9,000	Left cerebrum. Frontal lobe: Medial frontal gyrus	45
2	−4	16	34		Left cerebrum. Limbic lobe: Cingulate gyrus	
2	2	4	54		Right cerebrum. Frontal lobe: Superior frontal gyrus	
3	30	18	6	4,608	Right cerebrum. Sub‐lobar. Claustrum	34
3	42	16	6		Right cerebrum. Sub‐lobar: Insula	
3	42	24	6		Right cerebrum. Frontal lobe: Inferior frontal gyrus	
4	−8	46	8	4,504	Left cerebrum. Frontal lobe: Medial frontal gyrus	29
4	4	50	16		Right cerebrum. Frontal lobe: Medial frontal gyrus	
4	−4	38	14		Left cerebrum. Limbic lobe: Anterior cingulate	
4	−6	36	20		Left cerebrum. Limbic lobe: Anterior cingulate	
5	44	6	30	2,976	Right cerebrum. Frontal lobe: Inferior frontal gyrus	24
5	50	4	16		Right cerebrum. Frontal lobe: Inferior frontal gyrus	
6	22	42	20	2,448	Right cerebrum. Frontal lobe: Superior frontal gyrus	21
6	30	48	14		Right cerebrum. Frontal lobe: Superior frontal gyrus	
7	−46	4	34	2040	Left cerebrum. Frontal lobe: Precentral gyrus	19
8	−38	18	2	1864	Left cerebrum. Sub‐lobar: Insula	20

Abbreviations: Cluster #; cluster number; Vol, volume in mm^3^.

### 3.3 Step 2: Subtraction and conjunction analyses between pairs of SUD categories

**FIGURE 3:**
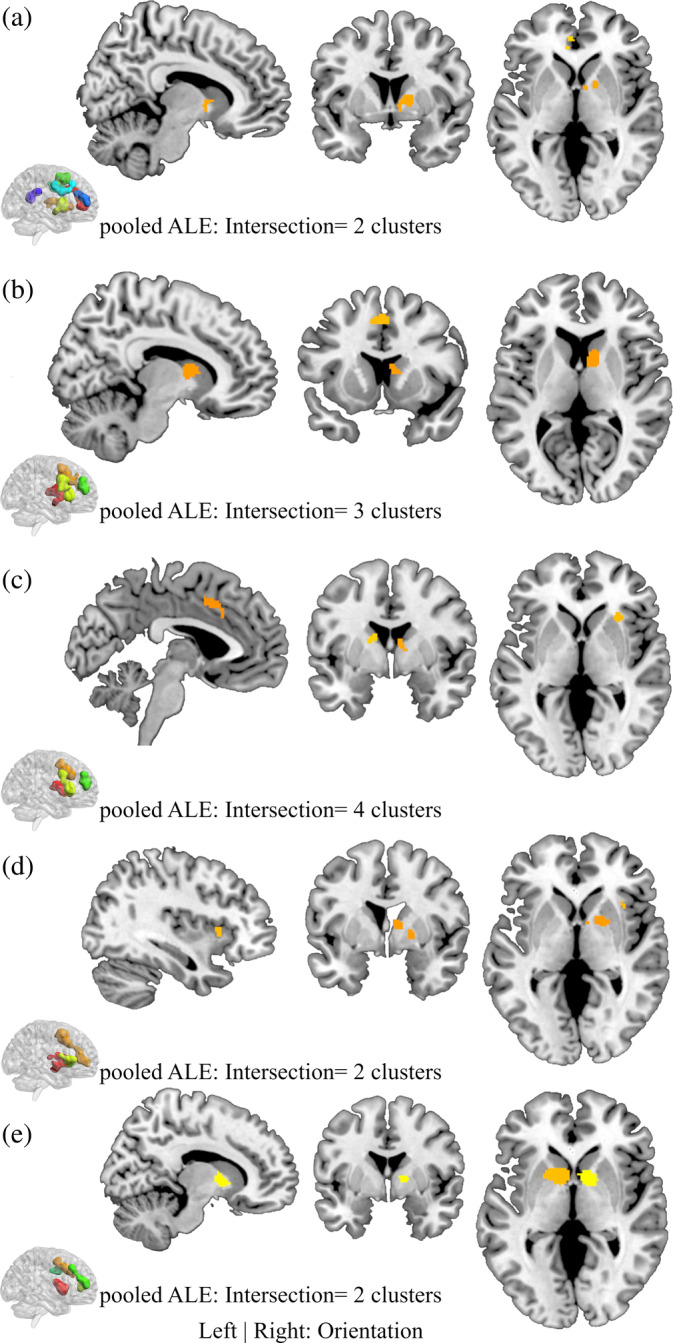
Conjunction analysis for each study pair. (a) Alcohol versus Cocaine, (b) Cannabis versus Cocaine, (c) Cannabis versus Nicotine, (d) Cocaine versus Nicotine, and (e) Nicotine versus Alcohol. Displayed at FWE < 0.05

The subtraction (direct voxel‐wise subtraction of ALE images) analyses aimed at determining differential neurofunctional alterations between the SUDs and revealed primarily differential neurofunctional alterations in frontal regions (details provided in Figure S1, Table S2). For instance, comparing alcohol with cannabis users revealed that the alcohol group shows greater alterations in the left middle frontal gyrus compared with the cannabis group that is characterized by pronounced alterations in the right caudate, right insula, right superior frontal gyrus, and right inferior frontal gyrus. In addition, nicotine associated changes are greater in the bilateral caudate and left anterior cingulate compared to cocaine and alcohol. The conjunction analysis aimed at determining overlapping foci of neurofunctional alterations between pairs of SUD categories as indicated in Step 2 in the method section. In the alcohol versus the cocaine group, eight clusters were obtained from the pooled foci results; we observe two clusters—one in the frontal gyrus and the other in the dorsal striatum (Figure 3a). Similarly, the contrast between cannabis and cocaine reveals four significant clusters, exhibiting an overlap of one cluster with the striatum (dorsal) and of two clusters with the frontal lobe (Superior Frontal Gyrus and Medial Frontal Gyrus; Figure 3b). There is no conjunction between alcohol and cannabis. Details of the other conjunction analyses are shown in Figure 3c–e and Table 3.

**TABLE 3:** Peak ALE coordinates for the paired conjunctions

Cluster #	x	y	z	Vol	Label	No. contributing studies
Conjunction: Alcohol versus cocaine						
1	14	6	4	592	Right cerebrum. Sub‐lobar: Putamen	3
1	8	4	−2		Right cerebrum. Sub‐lobar: Caudate	
2	−8	48	8	496	Left cerebrum. Frontal lobe: Medial frontal gyrus	3
2	−4	48	6		Left cerebrum. Limbic lobe: Anterior cingulate	
2	−4	44	2		Left cerebrum. Limbic lobe: Anterior cingulate	
2	−8	36	2		Left cerebrum. Limbic lobe: Anterior cingulate	
Conjunction: Cannabis versus cocaine						
1	10	6	10	1,000	Right cerebrum. Sub‐lobar: Caudate	8
2	−2	12	50	968	Left cerebrum. Frontal lobe: Superior frontal gyrus	8
2	−6	12	48		Left cerebrum. Frontal lobe: Superior frontal gyrus	
3	20	40	20	208	Right cerebrum. Frontal lobe: Medial frontal gyrus	3
3	18	48	24		Right cerebrum. Frontal lobe: Superior frontal gyrus	
Conjunction: Cannabis versus nicotine						
1	0	12	42	1,104	Left cerebrum. Frontal lobe: Medial frontal gyrus	12
2	8	6	10	1,024	Right cerebrum. Sub‐lobar: Caudate	7
3	30	18	8	1,008	Right cerebrum. Sub‐lobar: Claustrum	3
4	−12	−4	18	984	Left cerebrum. Sub‐lobar: Caudate	7
4	−12	6	12		Left cerebrum. Sub‐lobar: Caudate	
Conjunction: Cocaine versus nicotine						
1	10	6	10	2,088	Right cerebrum. Sub‐lobar: Caudate	9
1	20	4	0		Right cerebrum. Sub‐lobar: Putamen	
2	36	16	4	184	Right cerebrum. Sub‐lobar: Insula	
Conjunction: Nicotine versus alcohol						
1	−14	10	0	1,736	Left cerebrum. Sub‐lobar: Putamen	14
1	−6	8	−2	1,080	Left cerebrum. Sub‐lobar: Caudate	7
2	10	8	2		Right cerebrum. Sub‐lobar: Caudate	

Abbreviations: Cluster #; cluster number; Vol, volume in mm^3^.

### 3.4 Step 3: Post hoc analyses: Contribution of reward and cognitive domains and associations with duration of use

**FIGURE 4:**
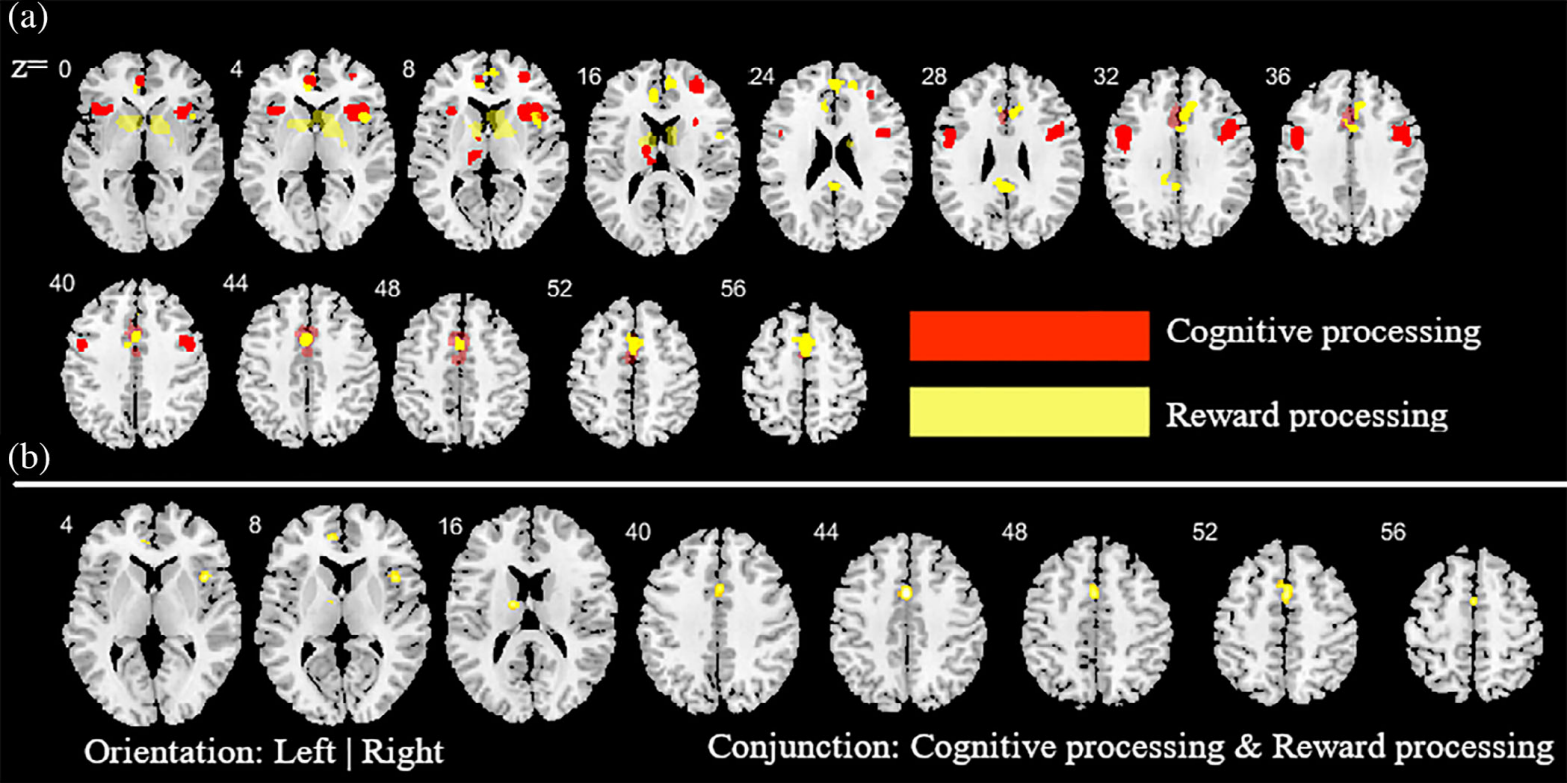
Additional analysis aimed at determining the contribution of the cognitive and reward‐based task paradigms for the main meta‐analysis across all substances. (a) ALE peak maps for the common (conjunction contrast) and differential (subtraction contrast) between the two types of task paradigms. Red (cognitive processing tasks > reward processing tasks); Yellow (reward processing tasks > cognitive processing tasks). (b) between the two types of task paradigm. Displayed at FWE < 0.05

**FIGURE 5:**
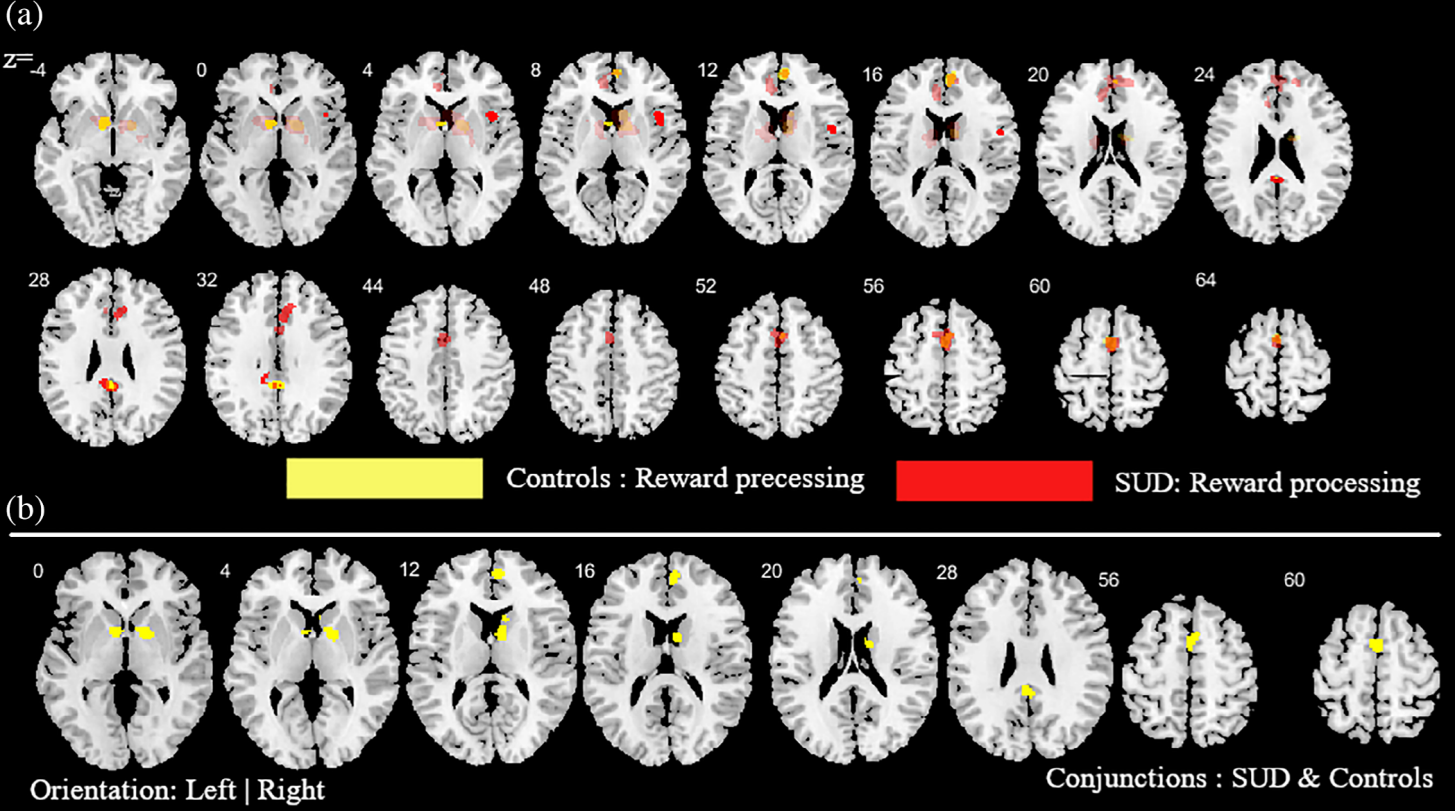
Additional analysis aimed at mapping alterations in SUD (across all substances) relative to controls as well as common activities between SUD and controls during reward‐related task paradigms. (a) Subtraction ALE comparing SUD and controls during reward task paradigms. Yellow (controls > SUD); Red (SUD > controls). (b) Conjunction ALE maps for reward‐related task paradigms between SUD and control participants. Displayed at FWE < 0.05

**FIGURE 6:**
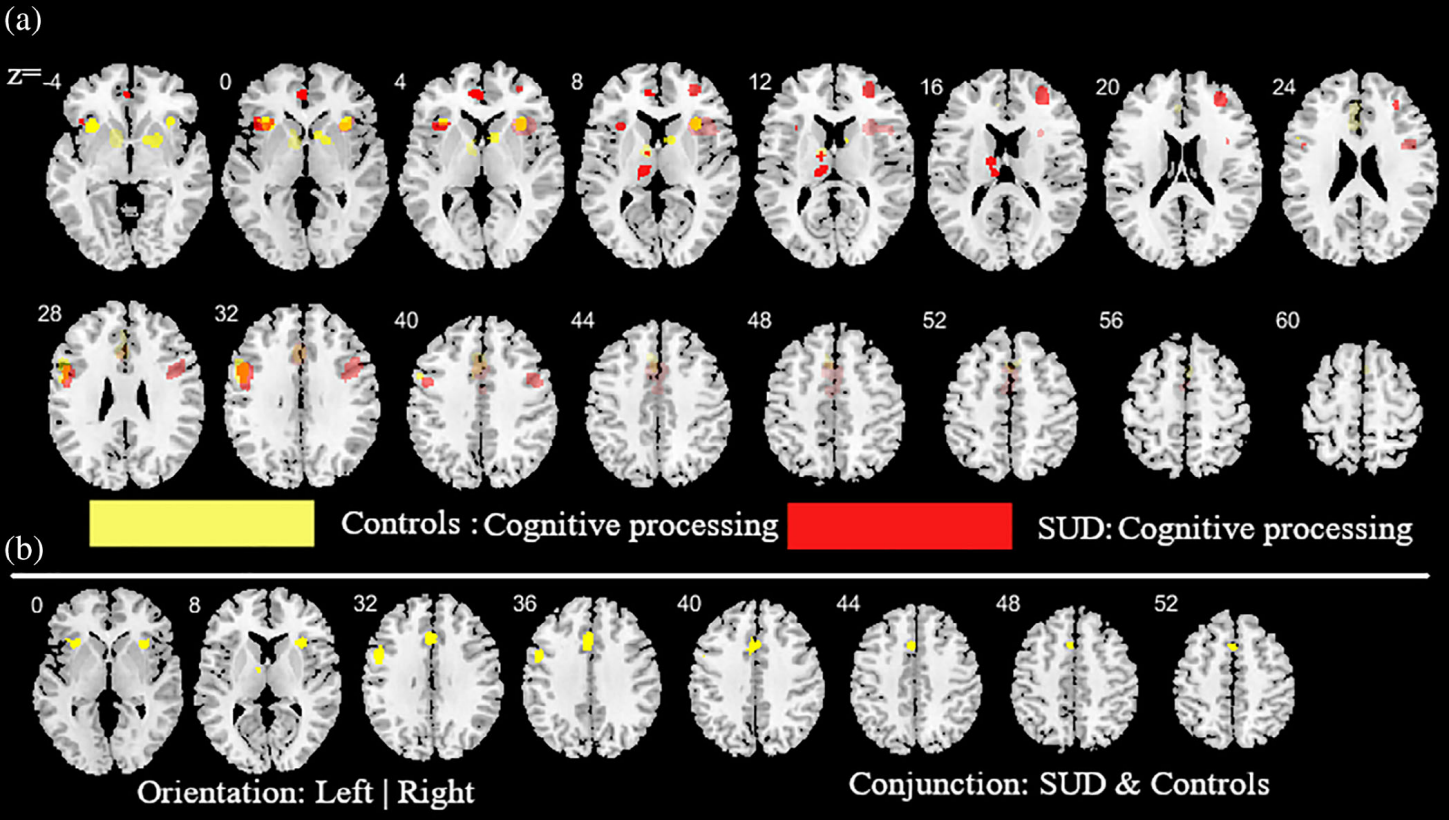
Additional analysis aimed at mapping alterations in SUD (across all substances) relative to controls as well as common activities between SUD and controls during cognitive task paradigms. (a) subtraction ALE comparing SUD and controls during cognitive task paradigms. Yellow (controls > SUD); Red (SUD > controls). (b) Conjunction ALE maps for cognitive task paradigms between SUD and control participants. Displayed at FWE < 0.05

**FIGURE 7:**
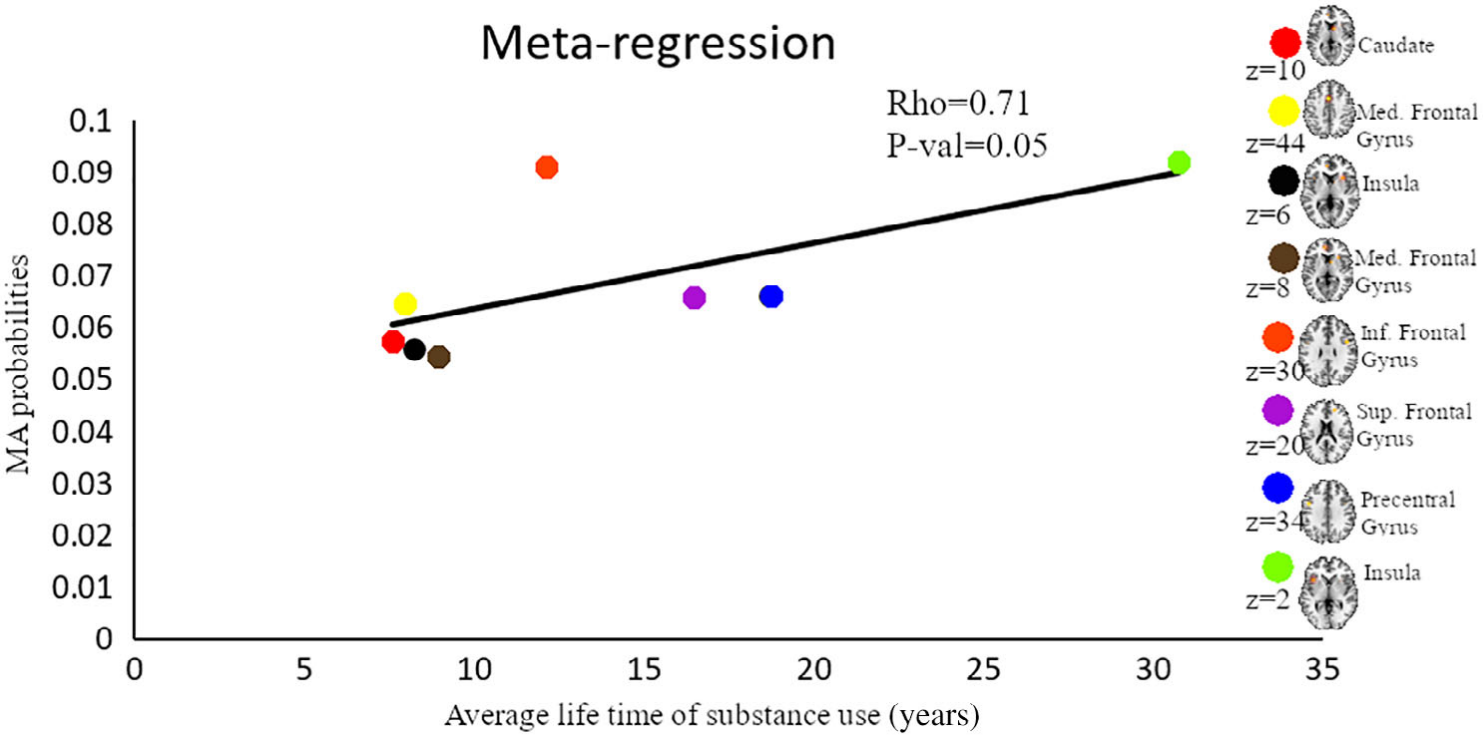
Results from the meta‐regression. Displayed are results from a meta‐regression between meta‐analytic probability values and duration of life‐time use of substance. Each color represents a cluster generated from the combined meta‐analysis of all substances. Brain map shows the location of maximum ALE with highest probability. Med, medial; Inf, Inferior; Sup, superior

To determine whether the employed task paradigms contributed to the identified neurofunctional alterations observed in the main meta‐analysis, interactions between the most commonly employed task paradigms were examined. Given that the majority of studies employed reward‐related and cognitive paradigms these domains were examined (given the low number of original studies that employed emotion processing paradigms these were not included). Consequently, a total of 39 reward‐related and 55 cognitive processing studies entered this analysis. Detailed results of this analysis are provided in Figures 4, 5, 6 (see also Tables S3 and S4 for detailed coordinate information). Briefly, the analysis reveals pronounced neurofunctional alterations in the frontal lobe during cognitive tasks in SUD. In addition, SUD exhibits stronger functional alterations in the reward system during reward‐related task paradigms, primarily in the dorsal striatum and frontal lobe regions involved in reward and salience processing. In addition, to determine whether different task‐paradigms employed may contribute to the neurofunctional differences and conjunctions we examined the contributions of the task paradigms to the corresponding analyses (see Figure S3 for subtraction analysis between the pair of SUDs). Using the Kruskal‐Wallis test, we did not find significant differences between the number of foci contributed by the category of task paradigm to the conjunction results (*χ*^2^ = 1.97, *p* = .37, see also Figure S2 for the percentage distribution of the task and foci). Together these additional analyses suggest that differences in the task paradigms are unlikely to bias the determination of the distinct and common neurofunctional alterations between the SUDs. Finally, a meta‐regression with the duration of use in years and the eight identified foci of the main meta‐regression across all substances was performed (number of studies with available data for duration *n* = 69). Findings from this exploratory analysis revealed that striatal and medial frontal regions involved in reward and value processing showed a higher probability to be identified in studies with participants with shorter duration of use while frontal regions engaged in regulatory and executive control such as the inferior, superior and precentral gyrus exhibited a higher probability to be identified in studies conducted in SUD samples with a longer duration of use (*p* = .05, rho = 0.71) (see Figure 7).

## 4 DISCUSSION

The present meta‐analytic approach employed a whole‐brain coordinate‐based meta‐analysis to determine shared and substance‐specific alterations in the most prevalent substance use disorders (alcohol, nicotine, cannabis, and cocaine) as determined in previous fMRI studies. Given that most previous studies could be classified in examining reward‐related functions or cognitive functions, we additionally examined domain‐specific alterations to determine whether dysregulations in distinct behavioral domains are neurally mediated by separable brain systems. In line with most overarching translational addiction models (e.g., [Everitt & Robbins, 2016; Koob & Volkow, 2016; Zilverstand et al., 2018]) the main meta‐analysis demonstrates robust functional alterations in frontostriatal regions, particularly dorsal striatal regions involved in habit formation and compulsive behavior, as well as prefrontal regions including anterior cingulate, inferior frontal and medial prefrontal regions critically engaged in executive control and behavioral regulation. Exploratory substance‐specific meta‐analyses furthermore reveal a consistent pattern of altered neural processing in striatal and prefrontal regions for the separate substances, with some evidence for less frontal impairments in nicotine addiction. The comparative analyses between the substances moreover indicate some evidence for differential effects in frontostriatal regions as well as limbic regions such as the ACC and the insular cortex. Further examining substance‐specific differences reveals that the experimental paradigm is independent of the observed activations except for alcohol versus cocaine and nicotine versus alcohol, these are mostly driven by reward‐based experiments. Cocaine and cannabis overlapped in the inferior frontal gyrus while alcohol‐related patterns showed no overlap with cannabis for the contrast between reward‐related and cognitive paradigms. Examining functional domain‐specific alterations across all substances shows that substance users demonstrate predominately striatal alterations during reward processes but frontal alterations during cognitive processes, suggesting that alterations in different behavioral domains are mediated by alterations in separable neural systems. Finally, an exploratory meta‐regression suggests higher meta‐analytic probabilities of neurofunctional alterations in reward‐related processing regions, particularly the striatum and medial frontal regions, in studies examining SUD subjects with a shorter duration of use, while frontal regions engaged in regulatory and executive control such as the inferior, superior and precentral gyrus exhibited a higher probability to be identified in studies conducted in SUD samples with a longer duration of use.

In general, findings from the present meta‐analysis confirmed the extensive animal and human literature suggesting a critical role of the frontostriatal circuits in addiction. Neuroadaptations in this circuitry have been associated with behavioral dysregulations in the domains of incentive salience and reward processing, habit formation and executive control (Everitt & Robbins, 2016; Koob & Volkow, 2016; Zilverstand et al., 2018) and may underpin the progressive loss of control that represents a key symptom across substance use disorders. In line with the key symptomatic deficits in salience/reward processing and executive control deficits in substance use disorders most previous studies employed corresponding task‐based paradigms examining associated neural processes. Comparing neural alterations in these domains across substances demonstrate stronger alterations in frontal regions during cognitive processes whereas alterations during reward/salience processing are neurally underpinned by stronger alterations in striatal regions and limbic regions, particularly the ACC. These findings resonate with the critical engagement of the frontal cortex in cognitive functions, including inhibitory control, decision making, and working memory which have been consistently found impaired in populations characterized by chronic substance use (Goldstein & Volkow, 2011; Morein‐Zamir & Robbins, 2015; Wesley & Bickel, 2014). The ventral striatum represents one of the most commonly identified regions showing alterations in previous meta‐analyses encompassing neuroimaging studies in addiction, including both drug‐related as well as nondrug related reward processing (Chase et al., 2011; Kühn & Gallinat, 2011). Together with the orbitofrontal cortex and the ACC the ventral striatum is engaged in evaluating the subjective value of stimuli in the environment (Zilverstand et al., 2018) and has been associated with impulsive choices and trait impulsivity (Barlow et al., 2018; Dalley & Robbins, 2017). Accordingly, alterations in this region may reflect adaptations in incentive‐based learning processes that promote exaggerated salience attributed to the drug as well as deficits in controlling impulsive behavior. The dorsal striatum, on the other hand, has been strongly associated with habit learning and the transition from reward‐driven to compulsive behavior in addiction (Vollstädt‐Klein et al., 2010; Zhou et al., 2018, 2019) and may promote the development of compulsive drug use in the context of progressive loss of behavioral control (Everitt & Robbins, 2013). Together, these findings emphasize that separable neural systems may mediate specific behavioral dysregulations and key diagnostic symptoms that characterize substance use disorders.

In line with the different neurobiological profiles of the substances and increasing evidence for substance‐specific alterations, the present meta‐analysis revealed evidence for differential alterations in the substance‐using populations. Alcohol use disorder was characterized by stronger alterations in frontal regions compared to the other three substances examined which may point to differential neurocognitive deficits in substance use disorders with alcohol use disorder being characterized by marked impairments in the domains of cognitive flexibility and attention (Fernández‐Serrano et al., 2011). In addition, reduced self‐control and the ability to obtain self‐regulation are linked with SUDs such as esaclating alcohol use, and other health‐threatening behaviors, thus, stronger self‐regulation moderates the usage of the substance (Neal & Carey, 2007; Wills, Ainette, Stoolmiller, Gibbons, & Shinar, 2008). Alcohol, for instance, has been shown to have a stronger effect in terms of self‐regulation (Quinn & Fromme, 2010) which is a primary indicator of prefrontal processes. For nicotine use disorder stronger alterations in striatal and insula regions, yet comparably fewer alterations in frontal regions were observed. These findings may underscore the high addictive potential of tobacco, with rather moderate cognitive impairments in tobacco users (Becker & Hurlemann, 2016) as well as an important role of the insula in nicotine addiction (Gaznick, Tranel, McNutt, & Bechara, 2014). Given the high prevalence of nicotine addiction across populations with substance use disorders (Agrawal, Budney, & Lynskey, 2012) these findings furthermore stress the importance to control for tobacco use in neuroimaging studies on addiction.

Moreover, overlapping alterations across the addictive disorders were observed in the dorsal striatum and the superior frontal gyrus.This may suggest an importnat role of the dorsal striatum engaged in associative learning, cognitive control, and decision‐making in addiction in contrast to conceptualizations that stress the important role of the ventral striatum such as reward and anticipation theories of addiction (Blum et al., 2000; Cloninger, 1991). Cannabis use is associated with greater alterations in frontal regions comparative to cocaine, suggesting that impaired executive control may be dominant in cannabis compared to cocaine use disorder which may be predominately driven by dysregulated reward anticipation. Long term cannabis use has been associated with dysfunctional frontal processes related to cognition such as response time to decision cues and verbal memory (Lundqvist, Jönsson, & Warkentin, 2001; Shrivastava, Johnston, & Tsuang, 2011) while cocaine use has been repeatedly associated with marked dysregulations in motivation and executive functions (Breiter et al., 1997; Luciana & Collins, 2012; Paulsen, Hallquist, Geier, & Luna, 2015). Moreover, alcohol and nicotine abuse shared common alterations in cortical regions and generally showed similar alterations to the other drugs, which may reflect the high rates of co‐abuse of nicotine and alcohol in many drug abusers (Cross, Lotfipour, & Leslie, 2017; Kohut, 2017). However, alcohol use was additionally characterized by greater alterations across limbic areas including the ACC suggesting stronger dysregulations in salience processing, reinforcement learning and decision making in contrast to nicotine which may predominately disrupt striatal reward‐related processes. Furthermore, as mentioned in the method section, we hypothesized that nicotine as often co‐abused substance across all other substances (Kohut, 2017) would share common functional alterations with the other substances. Remarkably, our results were consistent with the hypothesis but we additionally observed nicotine‐specific alterations in the thalamus that were not observed in the main meta‐analysis encompassing all drug classes. The thalamus exhibits particularly high expressions of nicotine‐sensitive receptors in the brain, which may partly contribute to the nicotine‐specific alterations observed in this region (Wonnacott, 1997; Zubieta et al., 2001). This nicotine‐specific finding may reflect that in addition to striatal reward‐related dysregulations nicotine use induced neuroadaptations in the thalamic circuit which may contribute to inhibition impairments observed in both, animal and human models of nicotine addiction (Huang, Mitchell, Haber, Alia‐Klein, & Goldstein, 2018). The thalamus may, therefore, specifically contribute to nicotine abuse.

Finally, results from an exploratory meta‐regression suggest that the meta‐analytic probability of the fronto‐striatal regions identified in the main meta‐analysis (across all substances) varies as a function of the duration of use. Specifically, striatal and frontal regions engaged in reward processing showed a high probability of being identified in studies conducted in SUD subjects with a comparably short duration of use, while frontal regions engaged in regulatory and executive control such as the inferior and superior frontal gyrus exhibited a higher probability of being identified in studies conducted in SUD subjects with a longer duration of use. Together these findings may suggest that reward‐related processing regions may become compromised during earlier stages of the addictive process while cognitive control regions become compromised during later stages. Moreover, the findings may suggest that the observed alterations—at least partly—represent consequences of continued substance use rather than stable predisposing alterations that precede the onset of drug use.

Although this meta‐analysis revealed task‐specific alterations between the various groups of studies, we could not conduct substance‐specific sub‐analyses comparing the different tasks within each substance group. This was due to the limited number of substance‐specific studies. The within‐group task‐based analysis would have thrown more light on the substance‐specific alterations for the individual substance use disorders. Furthermore, the reproducibility of common models for substance abuse in this meta‐analysis may also be related to selection and publication bias of studies. Moreover, despite our a priori hypothesis rooted in extensive animal models and human imaging studies the meta‐analysis was not pre‐registered. Finally, our analyses did not examine the direction of activation alterations between SUD subjects and controls and thus it cannot interfere whether the observed alterations represent hyper‐ or hypo‐activations in SUD.

## 5 CONCLUSION

Summarizing, our analysis reveals consistent results with convergent models from animal and human studies demonstrating that addiction is characterized by neural dysregulations in systems subserving salience/reward processes, habit learning, and executive control, including decision‐making and response inhibition, specifically the dorsal striatum and the prefrontal cortex. On the other hand, the present meta‐analytic approach allowed us to determine substance‐specific alterations in frontal, limbic, as well as insular regions, pointing to specific pathological alterations in addition to shared pathological pathways.

## CONFLICT OF INTEREST

The authors report no conflicts of interest.

## Supporting information


**Appendix S1:** Supporting infomation

**Figure S1** Subtraction analysis between pairs of studies.“>” the symbol indicates where ALE peaks are greater in one study compared to the other. Cluster forming (*p* < .001) and cluster‐level threshold (*p* < .05)

**Figure S2** Contributions to the conjunction between the two category of task paradigm, A) the Percentage distribution of each task paradigm to the conjunctions, B) the percentage distribution of foci to the conjunction. N; the number of foci per paradigm

**Figure S3** Comparing conjunction based on studies and tasks. Each row Figure label shows conjunction based on studies (see Figure 4 in the main paper) and row label 2 shows conjunction based on task respectively

## Data Availability

The data are available from the corresponding authors upon reasonable request.
