# Supplementary material for: Common and separable neural alterations in substance use disorders: A coordinate‐based meta‐analyses of functional neuroimaging studies in humans
Source: Hum Brain Mapp. 2020 Sep 10;41(16):4459–77. doi: 10.1002/hbm.25085 (PMC7555084; doi:10.1002/hbm.25085)
Supplement: Supplementary file 1 — Appendix S1: Supporting infomation [file HBM-41-4459-s002.docx]

**Common and separable neural alterations in substance use disorders: a coordinate-based meta-analyses of functional neuroimaging studies in humans**

Benjamin Klugah-Brown, PhD^1^ Xin Di, PhD^2^, Jana Zweerings, PhD^3,4^, Klaus Mathiak, PhD^3,4^, Benjamin Becker, PhD^1*^, Bharat Biswal, PhD^1,2*^

^1^ The Clinical Hospital of Chengdu Brain Science Institute, MOE Key Laboratory for Neuroinformation, Center for Information in Medicine, School of Life Science and Technology, University of Electronic Science and Technology of China, No.2006, Xiyuan Avenue, West Hi-Tech Zone, Chengdu, Sichuan 611731, China

^2^ Department of Biomedical Engineering, New Jersey Institute of Technology, 619 Fenster Hall, Newark, NJ 07102, USA

^3^ Department of Psychiatry, Psychotherapy and Psychosomatics, Faculty of Medicine, RWTH Aachen, Pauwelstrasse 30, 52074, Aachen, Germany

^4^ JARA Translational Brain Medicine, RWTH Aachen, Pauwelstrasse 30, 52074, Aachen, Germany

**Acknowledgments**

This work was supported by the National Key Research and Development Program of China (Grant No. 2018YFA0701400), National Natural Science Foundation of China (NSFC, No 91632117), and Science, Innovation and Technology Department of the Sichuan Province (2018JY0001).

**Conflict of interest**

The authors report no conflicts of interest.

***Corresponding authors**

Bharat Biswal

Email: [bbiswal@gmail.com](mailto:bbiswal@gmail.com)

Benjamin Becker

Email: [ben_becker@gmx.de](mailto:ben_becker@gmx.de)

**Supplementary material**


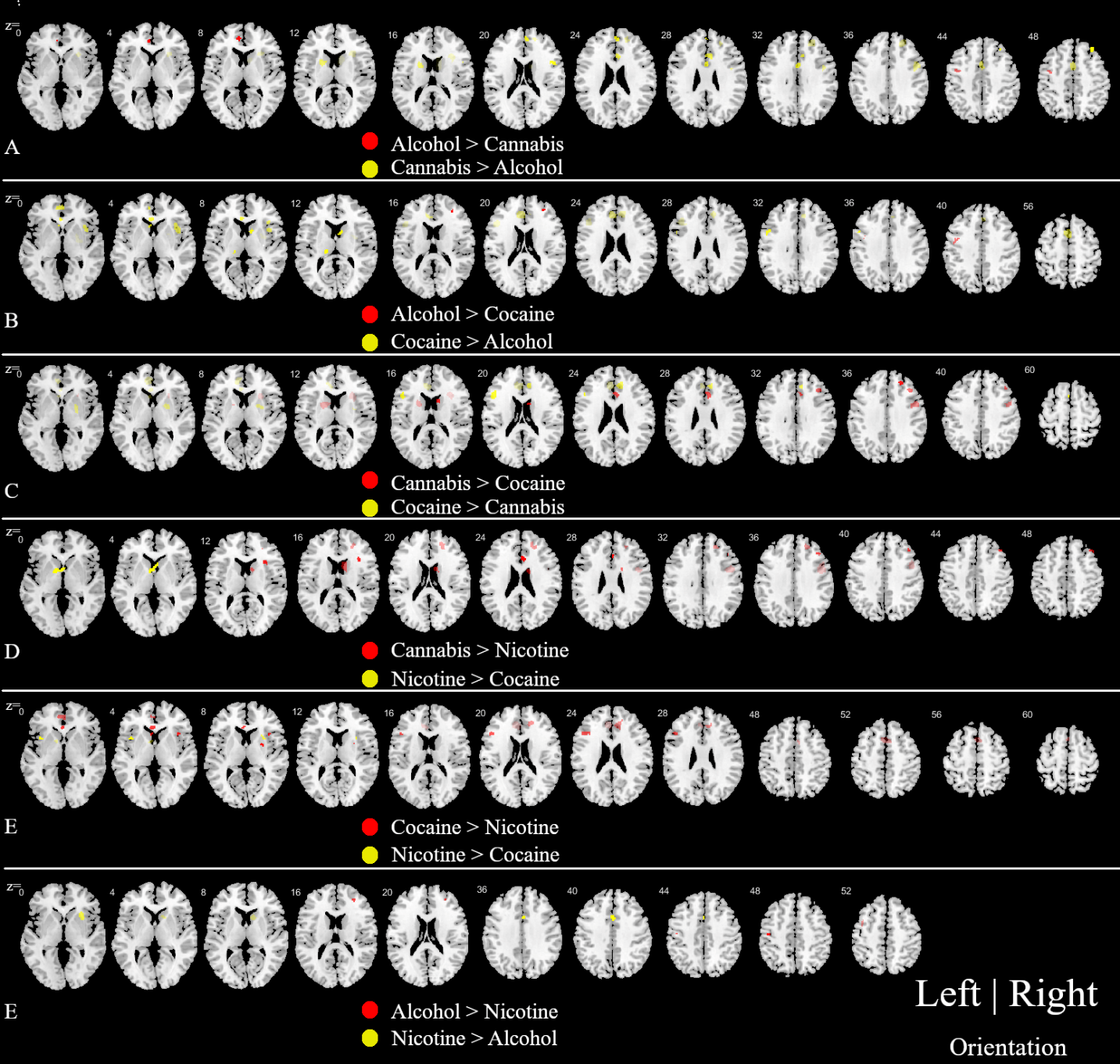


Figure S1 Subtraction analysis between pairs of studies.‘>’ the symbol indicates where ALE peaks are greater in one study compared to the other. Cluster forming (p<0.001) and cluster-level threshold (p<0.05)

Table 1 Detailed peak coordinates from each subtraction in the pairs ">" signifies greater than between the pairs

| Cluster # |  |  |  |  |  |  |
| --- | --- | --- | --- | --- | --- | --- |
| Alcohol > Cannabis | x | y | z | P | Z | Label |
| 1 | -40 | -2 | 46 | 0.01 | 2.326348 | Left Cerebrum. Frontal Lobe. Middle Frontal Gyrus |
| 2 | -8 | 46 | 6 | 0.013 | 2.226212 | Left Cerebrum. Frontal Lobe. Medial Frontal Gyrus. |
| Cannabis > Alcohol |  |  |  |  |  |  |
| 1 | 12.7 | 15 | 15.5 | 0 | 3.290527 | Right Cerebrum.Sub-lobar. Caudate |
| 1 | 12 | 6 | 18 | 0.002 | 2.878162 | Right Cerebrum.Sub-lobar. Caudate |
| 1 | 10 | 9 | 10 | 0.003 | 2.747781 | Right Cerebrum.Sub-lobar. Caudate. |
| 2 | 28 | 24 | 8 | 0.004 | 2.65207 | Right Cerebrum.Sub-lobar. Claustrum. |
| 2 | 30.3 | 24.3 | 6.5 | 0.002 | 2.878162 | Right Cerebrum.Sub-lobar. Insula. |
| 2 | 34 | 22.7 | 16.7 | 0.005 | 2.575829 | Right Cerebrum.Sub-lobar. Insula. |
| 2 | 34 | 18 | 18 | 0.006 | 2.512144 | Right Cerebrum.Sub-lobar. Insula. |
| 3 | 25.2 | 44.4 | 38 | 0 | 3.290527 | Right Cerebrum. Frontal Lobe. Superior Frontal Gyrus. |
| 3 | 23.2 | 46.3 | 32.7 | 0.002 | 2.878162 | Right Cerebrum. Frontal Lobe. Superior Frontal Gyrus. |
| 3 | 20.7 | 39.8 | 35.1 | 0.003 | 2.747781 | Right Cerebrum. Frontal Lobe. Superior Frontal Gyrus. |
| 3 | 14 | 52 | 22 | 0.009 | 2.365618 | Right Cerebrum. Frontal Lobe. Superior Frontal Gyrus. |
| 3 | 20 | 51 | 20 | 0.024 | 1.977369 | Right Cerebrum. Frontal Lobe. Superior Frontal Gyrus. |
| 4 | 46 | 4 | 36 | 0 | 3.290527 | Right Cerebrum. Frontal Lobe. Precentral Gyrus. |
| 4 | 46 | 10 | 38 | 0.002 | 2.878162 | Right Cerebrum. Frontal Lobe. Middle Frontal Gyrus. |
| 5 | 0 | 9 | 50 | 0.004 | 2.65207 | Left Cerebrum. Frontal Lobe. Superior Frontal Gyrus. |
| 6 | -18 | 6.7 | 15.3 | 0.007 | 2.457264 | Left Cerebrum.Sub-lobar. Caudate. |
| 6 | -22 | 12 | 16 | 0.008 | 2.408916 | Left Cerebrum.Sub-lobar. Claustrum. |
| 6 | -22.7 | 8 | 15.3 | 0.009 | 2.365618 | Left Cerebrum.Sub-lobar. Putamen |
| 7 | 8 | 22 | 24 | 0 | 3.290527 | Right Cerebrum. Limbic Lobe. Anterior Cingulate. |
| 8 | 2 | 5 | 33 | 0.005 | 2.575829 | Right Cerebrum. Limbic Lobe. Cingulate Gyrus. |
| 9 | 6 | 52 | 24 | 0.001 | 3.090232 | Right Cerebrum. Frontal Lobe. Superior Frontal Gyrus. |
| 10 | 44 | 12 | 22 | 0.024 | 1.977369 | Right Cerebrum. Frontal Lobe. Inferior Frontal Gyrus. |
| 10 | 52 | 10 | 18 | 0.036 | 1.799118 | Right Cerebrum. Frontal Lobe. Inferior Frontal Gyrus. |
| 10 | 52 | 6 | 22 | 0.042 | 1.727934 | Right Cerebrum. Frontal Lobe. Inferior Frontal Gyrus. |
| 11 | 32 | 38 | 50 | 0.001 | 3.090232 | Right Cerebrum. Frontal Lobe. Middle Frontal Gyrus. |
| 11 | 28 | 36 | 48 | 0.011 | 2.290368 | Right Cerebrum. Frontal Lobe. Middle Frontal Gyrus. |
| 11 | 32 | 34 | 50 | 0.012 | 2.257129 | Right Cerebrum. Frontal Lobe. Superior Frontal Gyrus. |
| Alcohol > Cocaine |  |  |  |  |  |  |
| 1 | -40 | 0 | 40 | 0.029 | 1.895698 | Left Cerebrum. Frontal Lobe. Precentral Gyrus. |
| 2 | 30 | 45 | 19 | 0.03 | 1.880794 | Right Cerebrum. Frontal Lobe. Middle Frontal Gyrus. |
| Cocaine > Alcohol |  |  |  |  |  |  |
| 1 | 30.8 | -3.5 | -1.5 | 0 | 3.290527 | Right Cerebrum.Sub-lobar. Putamen |
| 1 | 19.6 | -10.8 | -11.6 | 0.003 | 2.747781 | Right Cerebrum. Limbic Lobe. Parahippocampal Gyrus. |
| 2 | -45.2 | 25 | 23.3 | 0.001 | 3.090232 | Left Cerebrum. Frontal Lobe. Middle Frontal Gyrus. |
| 2 | -46.7 | 25.3 | 18 | 0.001 | 3.090232 | Left Cerebrum. Frontal Lobe. Inferior Frontal Gyrus. |
| 2 | -36 | 20 | 28 | 0.018 | 2.096927 | Left Cerebrum. Frontal Lobe. Middle Frontal Gyrus. |
| 3 | 6 | 35 | 39 | 0.002 | 2.878162 | Right Cerebrum. Frontal Lobe. Medial Frontal Gyrus. |
| 3 | 8.7 | 38 | 36.7 | 1 | 0 | Right Cerebrum. Frontal Lobe. Medial Frontal Gyrus. |
| 3 | 14 | 38 | 26 | 0.006 | 2.512144 | Right Cerebrum. Frontal Lobe. Medial Frontal Gyrus. |
| 3 | 0 | 34 | 42 | 0.007 | 2.457264 | Left Cerebrum. Frontal Lobe. Superior Frontal Gyrus. |
| 3 | 8 | 38 | 24 | 0.011 | 2.290368 | Right Cerebrum. Limbic Lobe. Anterior Cingulate. |
| 4 | -11.2 | 38.4 | 20 | 0 | 3.290527 | Left Cerebrum. Frontal Lobe. Medial Frontal Gyrus. |
| 4 | -4.5 | 37.5 | 20.5 | 0.001 | 3.090232 | Left Cerebrum. Limbic Lobe. Anterior Cingulate. |
| 5 | -1.5 | 11 | 55 | 0 | 3.290527 | Left Cerebrum. Frontal Lobe. Superior Frontal Gyrus. |
| 5 | -2.7 | 16 | 57.3 | 0.001 | 3.090232 | Left Cerebrum. Frontal Lobe. Superior Frontal Gyrus. |
| 6 | 40 | 12 | 1 | 0.004 | 2.65207 | Right Cerebrum.Sub-lobar. Insula. |
| 6 | 39 | 17 | 3 | 0.01 | 2.326348 | Right Cerebrum.Sub-lobar. Insula. |
| 6 | 40 | 28 | 8 | 0.008 | 2.408916 | Right Cerebrum. Frontal Lobe. Inferior Frontal Gyrus. |
| 7 | -5 | 50 | -2 | 0.008 | 2.408916 | Left Cerebrum. Limbic Lobe. Anterior Cingulate. |
| 8 | -4 | 30 | 8 | 0.004 | 2.65207 | Left Cerebrum. Limbic Lobe. Anterior Cingulate. |
| 8 | -2 | 28 | 4 | 0.007 | 2.457264 | Left Cerebrum. Limbic Lobe. Anterior Cingulate. |
| 8 | -8 | 20 | 2 | 0.008 | 2.408916 | Left Cerebrum.Sub-lobar. Caudate |
| 8 | -4 | 24 | 2 | 0.01 | 2.326348 | Left Cerebrum.Sub-lobar. Caudate |
| 9 | -48 | 12 | 32 | 0.011 | 2.290368 | Left Cerebrum. Frontal Lobe. Middle Frontal Gyrus. |
| 10 | 12 | 12 | 10 | 0.024 | 1.977369 | Right Cerebrum.Sub-lobar. Caudate. |
| 10 | 8 | 8 | 12 | 0.025 | 1.959964 | Right Cerebrum.Sub-lobar. Caudate. |
| 11 | -16 | -24 | 10 | 0.019 | 2.074855 | Left Cerebrum.Sub-lobar. Thalamus. |
| Cannabis > Cocaine |  |  |  |  |  |  |
| 1 | 25 | 20 | 8 | 0 | 3.290527 | Right Cerebrum.Sub-lobar. Claustrum. |
| 1 | 26 | 20 | 2 | 0.007 | 2.457264 | Right Cerebrum.Sub-lobar. Claustrum. |
| 2 | -22 | 2 | 12 | 0.008 | 2.408916 | Left Cerebrum.Sub-lobar. Putamen |
| 2 | -16.7 | 5.3 | 15.3 | 0.011 | 2.290368 | Left Cerebrum.Sub-lobar. Caudate. |
| 3 | 48 | 8 | 36 | 0.007 | 2.457264 | Right Cerebrum. Frontal Lobe. Middle Frontal Gyrus. |
| 3 | 42 | 2 | 38 | 0.011 | 2.290368 | Right Cerebrum. Frontal Lobe. Middle Frontal Gyrus. |
| 4 | 42 | 30 | 42 | 0.003 | 2.747781 | Right Cerebrum. Frontal Lobe. Middle Frontal Gyrus. |
| 4 | 40 | 32 | 46 | 0.005 | 2.575829 | Right Cerebrum. Frontal Lobe. Middle Frontal Gyrus. |
| 4 | 44 | 30 | 46 | 0.008 | 2.408916 | Right Cerebrum. Frontal Lobe. Middle Frontal Gyrus. |
| 4 | 36 | 30 | 32 | 0.011 | 2.290368 | Right Cerebrum. Frontal Lobe. Middle Frontal Gyrus. |
| 5 | 6 | 20 | 24 | 0.003 | 2.747781 | Right Cerebrum. Limbic Lobe. Anterior Cingulate. |
| 6 | 14 | 10 | 18 | 0.004 | 2.65207 | Right Cerebrum.Sub-lobar. Caudate. |
| 7 | 22 | 44 | 38 | 0.014 | 2.197286 | Right Cerebrum. Frontal Lobe. Superior Frontal Gyrus. |
| 7 | 24 | 40 | 38 | 0.015 | 2.17009 | Right Cerebrum. Frontal Lobe. Superior Frontal Gyrus. |
| 8 | -2 | 10 | 44 | 0.015 | 2.17009 | Left Cerebrum. Frontal Lobe. Medial Frontal Gyrus. |
| Cocaine > Cannabis |  |  |  |  |  |  |
| 1 | -4.8 | 35.6 | 21.1 | 0 | 3.290527 | Left Cerebrum. Limbic Lobe. Anterior Cingulate. |
| 1 | -9.4 | 39.5 | 12.2 | 0.003 | 2.747781 | Left Cerebrum. Limbic Lobe. Anterior Cingulate. |
| 1 | -8.7 | 34.7 | 4.7 | 0.141 | 0 | Left Cerebrum. Limbic Lobe. Anterior Cingulate. |
| 1 | -6 | 46 | -6 | 0.021 | 2.03352 | Left Cerebrum. Limbic Lobe. Anterior Cingulate. |
| 2 | 24 | 2 | 4 | 0.002 | 2.878162 | Right Cerebrum.Sub-lobar. Putamen |
| 2 | 24 | -8 | -10 | 0.007 | 2.457264 | Right Cerebrum.Sub-lobar. Amygdala |
| 2 | 26 | -6 | -2 | 0.023 | 1.995393 | Right Cerebrum.Sub-lobar. Putamen |
| 3 | 9.7 | 36 | 22.7 | 0.003 | 2.747781 | Right Cerebrum. Limbic Lobe. Anterior Cingulate. |
| 4 | -50 | 24 | 20 | 0.005 | 2.575829 | Left Cerebrum. Frontal Lobe. Inferior Frontal Gyrus. |
| 4 | -49.4 | 18 | 17.7 | 0.006 | 2.512144 | Left Cerebrum. Frontal Lobe. Inferior Frontal Gyrus. |
| 5 | -8 | 10 | 56 | 0.01 | 2.326348 | Left Cerebrum. Frontal Lobe. Superior Frontal Gyrus. |
| 5 | -5 | 15 | 57 | 0.02 | 2.053749 | Left Cerebrum. Frontal Lobe. Superior Frontal Gyrus. |
| Cannabis > Nicotine |  |  |  |  |  |  |
| 1 | 44.1 | 4.1 | 36.1 | 0 | 3.290527 | Right Cerebrum. Frontal Lobe. Precentral Gyrus. |
| 1 | 50 | 0 | 31 | 0.001 | 3.090232 | Right Cerebrum. Frontal Lobe. Precentral Gyrus. |
| 2 | 22 | 42 | 20 | 0.006 | 2.512144 | Right Cerebrum. Frontal Lobe. Superior Frontal Gyrus. |
| 2 | 24 | 40 | 16 | 0.008 | 2.408916 | Right Cerebrum. Frontal Lobe. Medial Frontal Gyrus. |
| 2 | 20 | 44 | 38 | 0.011 | 2.290368 | Right Cerebrum. Frontal Lobe. Superior Frontal Gyrus. |
| 2 | 24 | 38 | 34 | 0.014 | 2.197286 | Right Cerebrum. Frontal Lobe. Middle Frontal Gyrus. |
| 2 | 24 | 40 | 30 | 0.018 | 2.096927 | Right Cerebrum. Frontal Lobe. Superior Frontal Gyrus. |
| 3 | 10 | 8 | 20.7 | 0.004 | 2.65207 | Right Cerebrum.Sub-lobar. Caudate. |
| 3 | 12 | 13 | 18 | 0.003 | 2.747781 | Right Cerebrum.Sub-lobar. Caudate. |
| 3 | 18 | 2 | 26 | 0.008 | 2.408916 | Right Cerebrum.Sub-lobar. Caudate. |
| 4 | 41 | 30.5 | 46.5 | 0.019 | 2.074855 | Right Cerebrum. Frontal Lobe. Middle Frontal Gyrus. |
| 4 | 42 | 32 | 38 | 0.014 | 2.197286 | Right Cerebrum. Frontal Lobe. Middle Frontal Gyrus. |
| 5 | 36 | 20 | 14 | 0.005 | 2.575829 | Right Cerebrum.Sub-lobar. Insula. |
| 6 | 6 | 18 | 24 | 0 | 3.290527 | Right Cerebrum. Limbic Lobe. Anterior Cingulate. |
| 6 | 2 | 14 | 26 | 0.039 | 1.76241 | Right Cerebrum. Limbic Lobe. Cingulate Gyrus. |
| Nicotine > Cannabis |  |  |  |  |  |  |
| 1 | -2 | 6 | 2 | 0.004 | 2.65207 | Left Cerebrum.Sub-lobar. Caudate. |
| 1 | 0 | 6 | -2 | 0.005 | 2.575829 | Left Cerebrum. Limbic Lobe. Anterior Cingulate. |
| 1 | -8 | 4 | -2 | 0.008 | 2.408916 | Left Cerebrum.Sub-lobar. Caudate. |
| 1 | 4 | 14 | 2 | 0.01 | 2.326348 | Right Cerebrum.Sub-lobar. Caudate. |
| 1 | -12 | 4 | -2 | 0.012 | 2.257129 | Left Cerebrum.Sub-lobar. Lateral Globus Pallidus |
| Cocaine > Nicotine |  |  |  |  |  |  |
| 1 | -6.7 | 37.5 | 20.9 | 0 | 3.290527 | Left Cerebrum. Limbic Lobe. Anterior Cingulate. |
| 1 | 0 | 34.7 | 23.3 | 0.001 | 3.090232 | Left Cerebrum. Limbic Lobe. Anterior Cingulate. |
| 2 | 4.7 | 16 | 52.7 | 0.003 | 2.747781 | Right Cerebrum. Frontal Lobe. Superior Frontal Gyrus. |
| 2 | 3 | 14 | 56 | 0.002 | 2.878162 | Right Cerebrum. Frontal Lobe. Superior Frontal Gyrus. |
| 3 | 22 | 41 | 20 | 0 | 3.290527 | Right Cerebrum. Frontal Lobe. Medial Frontal Gyrus. |
| 3 | 16 | 40 | 24 | 0.006 | 2.512144 | Right Cerebrum. Frontal Lobe. Medial Frontal Gyrus |
| 3 | 7 | 35 | 25 | 0.015 | 2.17009 | Right Cerebrum. Limbic Lobe. Anterior Cingulate. |
| 3 | 14 | 34 | 26 | 0.01 | 2.326348 | Right Cerebrum. Frontal Lobe. Medial Frontal Gyrus. |
| 4 | 2 | 44 | -2 | 0.005 | 2.575829 | Right Cerebrum. Limbic Lobe. Anterior Cingulate. |
| 4 | -6 | 48 | 0 | 0.006 | 2.512144 | Left Cerebrum. Frontal Lobe. Medial Frontal Gyrus. |
| 4 | -4 | 44 | -4 | 0.009 | 2.365618 | Left Cerebrum. Limbic Lobe. Anterior Cingulate. |
| 5 | -46.7 | 24 | 25.3 | 0.015 | 2.17009 | Left Cerebrum. Frontal Lobe. Middle Frontal Gyrus. |
| 6 | -4 | 26 | 2 | 0.003 | 2.747781 | Left Cerebrum. Limbic Lobe. Anterior Cingulate. |
| 6 | -6 | 20 | 4 | 0.011 | 2.290368 | Left Cerebrum.Sub-lobar. Caudate |
| 7 | 38 | 20 | 4 | 0.011 | 2.290368 | Right Cerebrum.Sub-lobar. Insula. |
| 8 | 26 | 2 | 8 | 0.032 | 1.85218 | Right Cerebrum.Sub-lobar. Putamen |
| Nicotine > Cocaine |  |  |  |  |  |  |
| 1 | -12 | 8 | 0 | 0.027 | 1.926837 | Left Cerebrum.Sub-lobar. Lateral Globus Pallidus |
| 2 | 30 | 14 | 10 | 0.019 | 2.074855 | Right Cerebrum.Sub-lobar. Claustrum. |
| 3 | -38 | 12 | 2 | 0.008 | 2.408916 | Left Cerebrum.Sub-lobar. Insula. |
| Alcohol > Nicotine |  |  |  |  |  |  |
| 1 | -40 | -2 | 52 | 0.027 | 1.926837 | Left Cerebrum. Frontal Lobe. Middle Frontal Gyrus. |
| 1 | -40 | 6 | 52 | 0.039 | 1.76241 | Left Cerebrum. Frontal Lobe. Middle Frontal Gyrus. |
| 2 | 28 | 44 | 18 | 0.019 | 2.074855 | Right Cerebrum. Frontal Lobe. Superior Frontal Gyrus. |
| 3 | -45 | -14 | 47 | 0.014 | 2.197286 | Left Cerebrum. Frontal Lobe. Precentral Gyrus. |
| Nicotine > Alcohol |  |  |  |  |  |  |
| 1 | 14 | 16 | 8 | 0.003 | 2.747781 | Right Cerebrum.Sub-lobar. Caudate. |
| 2 | 34 | 16 | -4 | 0.015 | 2.17009 | Right Cerebrum.Sub-lobar. Inferior Frontal Gyrus. |
| 2 | 26 | 22 | 0 | 0.02 | 2.053749 | Right Cerebrum.Sub-lobar. Claustrum. |
| 2 | 30 | 22 | -2 | 0.021 | 2.03352 | Right Cerebrum.Sub-lobar. Insula. |
| 3 | 0 | 14 | 40 | 0.016 | 2.144411 | Left Cerebrum. Limbic Lobe. Cingulate Gyrus. |
| 3 | 2 | 10 | 36 | 0.019 | 2.074855 | Right Cerebrum. Limbic Lobe. Cingulate Gyrus. |
|  |  |  |  |  |  |  |

***postHoc analysis***

**Figure 2** shows a side-by-side visual comparison between ALE results due to the studies (labels A1-D1) and task experiments (A2-D2), we found no overlap for nicotine versus alcohol for the task paradigms. **Table 3** shows the detailed coordinate information for the task-based conjunction.


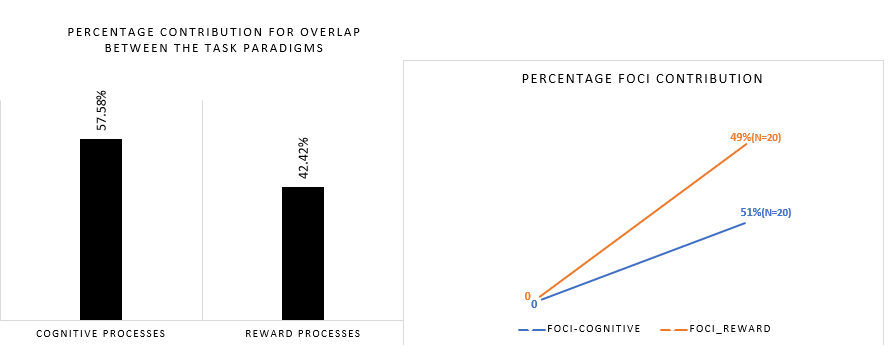


Figure S2 Contributions to the conjuction between the two category of task paradigm, A) the Percentage distribution of each task paradigm to the conjunctions, B) the percentage distribution of foci to the conjunction. N; the number of foci per paradigm


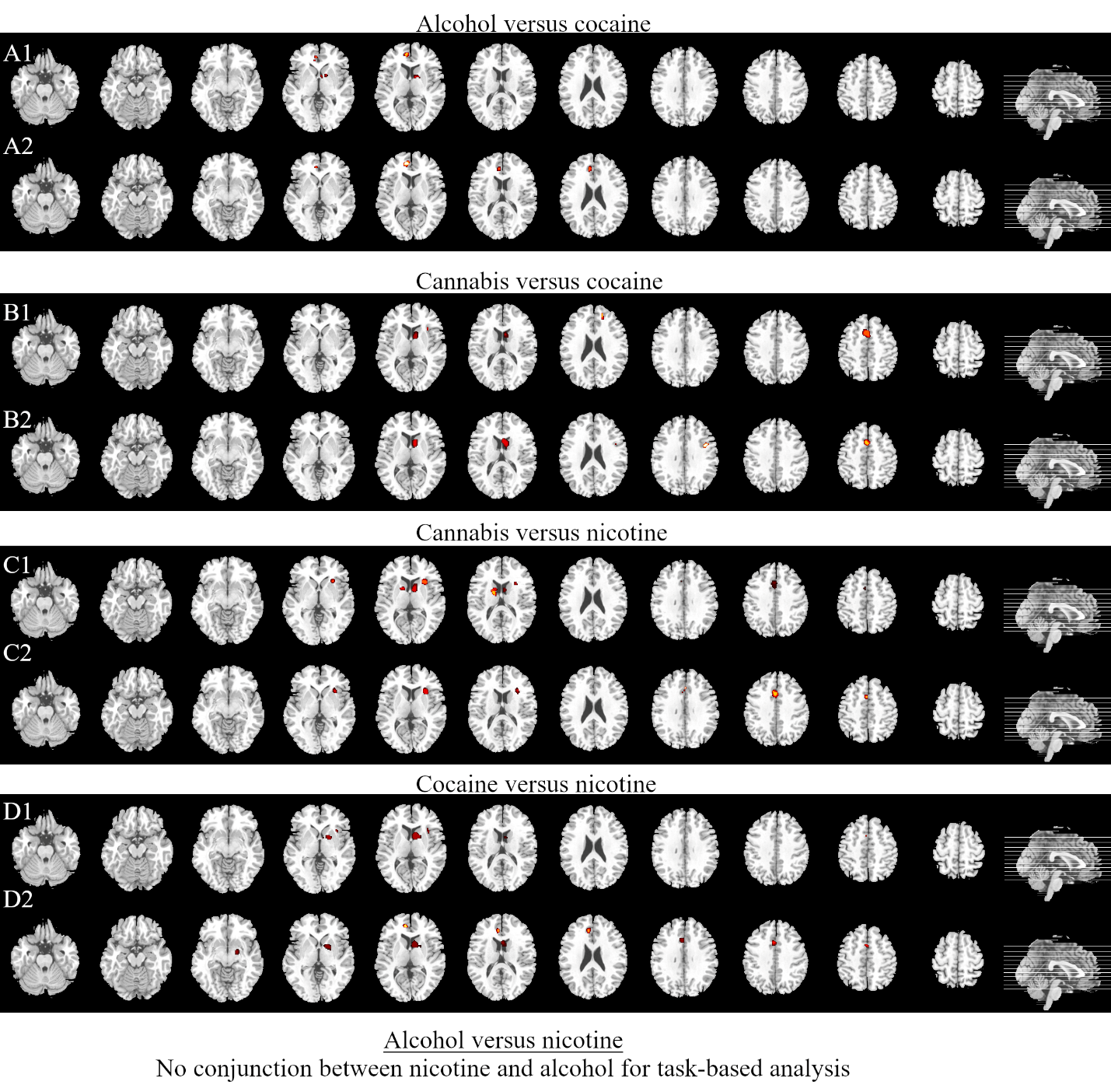


Figure S3 Comparing conjunction based on studies and tasks. Each row Figure label shows conjunction based on studies (see Figure 4 in the main paper) and row label 2 shows conjunction based on task respectively

Table 2 Detail coordinates forming the conjunction based on tasks. See the main paper Table 3 for studies-based conjunction.

| Cluster # | x | y | z | ALE | Label |
| --- | --- | --- | --- | --- | --- |
| A2: Alcohol versus cocaine | | | | | |
| 1 | -8 | 38 | 20 | 0.026185 | Left Cerebrum. Limbic Lobe. Anterior Cingulate. |
| 2 | -8 | 48 | 6 | 0.024981 | Left Cerebrum. Frontal Lobe. Medial Frontal Gyrus. |
| 2 | -6 | 40 | 0 | 0.017812 | Left Cerebrum. Limbic Lobe. Anterior Cingulate. |
| B2: Cannabis versus cocaine | | | | | |
| Cluster # | x | y | z |  | Label |
| 1 | 10 | 8 | 12 | 0.040678 | Right Cerebrum.Sub-lobar. Caudate. |
| 2 | 2 | 10 | 52 | 0.024521 | Right Cerebrum. Frontal Lobe. Superior Frontal Gyrus. |
| 3 | 44 | 4 | 34 | 0.022892 | Right Cerebrum. Frontal Lobe. Precentral Gyrus. |
| 3 | 42 | 2 | 30 | 0.02274 | Right Cerebrum. Frontal Lobe. Precentral Gyrus. |
| C2: Cannabis versus nicotine | | | | | |
| Cluster # | x | y | z |  | Label |
| 1 | 30 | 20 | 8 | 0.030983 | Right Cerebrum.Sub-lobar. Insula. |
| 2 | 0 | 8 | 44 | 0.03443 | Left Cerebrum. Frontal Lobe. Medial Frontal Gyrus. |
| 2 | -4 | 20 | 34 | 0.019912 | Left Cerebrum. Frontal Lobe. Cingulate Gyrus. |
| 3 | 2 | 26 | 30 | 0.016228 | Right Cerebrum. Limbic Lobe. Cingulate Gyrus. |
| D2: Cocaine versus nicotine | | | | | |
| Cluster # | x | y | z |  | Label |
| 1 | 10 | 8 | 8 | 0.02624 | Right Cerebrum.Sub-lobar. Caudate. |
| 1 | 20 | 4 | -2 | 0.025724 | Right Cerebrum.Sub-lobar. Lentiform Nucleus. Putamen |
| 1 | 20 | -6 | -8 | 0.023849 | Right Cerebrum.Sub-lobar. Lentiform Nucleus. Globus Pallidus |
| 2 | -4 | 18 | 32 | 0.023704 | Left Cerebrum. Limbic Lobe. Cingulate Gyrus. |
| 2 | 0 | 10 | 44 | 0.021835 | Left Cerebrum. Frontal Lobe. Medial Frontal Gyrus. |
| 2 | -2 | 12 | 52 | 0.019066 | Left Cerebrum. Frontal Lobe. Superior Frontal Gyrus. |
| 3 | -6 | 38 | 20 | 0.025814 | Left Cerebrum. Limbic Lobe. Anterior Cingulate. |
| 4 | -8 | 46 | 8 | 0.023994 | Left Cerebrum. Frontal Lobe. Medial Frontal Gyrus. |
| 5 | -6 | 38 | 4 | 0.016629 | Left Cerebrum. Limbic Lobe. Anterior Cingulate. |

Table 3 Detail peak cluster information for differences and conjunction between cognitive task and reward task for all substances

| Cluster # | x | y | z | ALE | Label |
| --- | --- | --- | --- | --- | --- |
| Cognitive processing > Reward reward processing |  |  |  |  |  |
| 1 | -2 | 10 | 44 | 0.053965 | Left Cerebrum. Frontal Lobe. Medial Frontal Gyrus. |
| 1 | -4 | 16 | 34 | 0.043687 | Left Cerebrum. Limbic Lobe. Cingulate Gyrus. |
| 1 | -2 | -4 | 52 | 0.040893 | Left Cerebrum. Frontal Lobe. Medial Frontal Gyrus. |
| 1 | 0 | 26 | 32 | 0.035746 | Left Cerebrum. Limbic Lobe. Cingulate Gyrus. |
| 1 | 2 | -6 | 42 | 0.035104 | Right Cerebrum. Limbic Lobe. Cingulate Gyrus. |
| 2 | 32 | 18 | 4 | 0.043585 | Right Cerebrum.Sub-lobar. Insula. |
| 2 | 32 | 18 | 8 | 0.041755 | Right Cerebrum.Sub-lobar. Insula. |
| 2 | 38 | 16 | 6 | 0.041452 | Right Cerebrum.Sub-lobar. Insula. |
| 2 | 48 | 16 | 10 | 0.03292 | Right Cerebrum. Frontal Lobe. Inferior Frontal Gyrus. |
| 3 | 44 | 8 | 30 | 0.038719 | Right Cerebrum. Frontal Lobe. Inferior Frontal Gyrus. |
| 3 | 48 | 0 | 38 | 0.034591 | Right Cerebrum. Frontal Lobe. Middle Frontal Gyrus. |
| 4 | -46 | 4 | 34 | 0.052699 | Left Cerebrum. Frontal Lobe. Precentral Gyrus. |
| 5 | 30 | 48 | 14 | 0.047302 | Right Cerebrum. Frontal Lobe. Superior Frontal Gyrus. |
| 6 | -40 | 22 | 0 | 0.036538 | Left Cerebrum. Frontal Lobe. Inferior Frontal Gyrus. |
| 6 | -32 | 20 | 6 | 0.035358 | Left Cerebrum.Sub-lobar. Insula. |
| 7 | -12 | -20 | 8 | 0.038503 | Left Cerebrum.Sub-lobar. Thalamus. |
| 7 | -12 | -8 | 14 | 0.030863 | Left Cerebrum.Sub-lobar. Thalamus. |
| 8 | -2 | 46 | 0 | 0.033395 | Left Cerebrum. Limbic Lobe. Anterior Cingulate. |
| 8 | -8 | 48 | 6 | 0.033241 | Left Cerebrum. Frontal Lobe. Medial Frontal Gyrus. |
| Reward processing > Cognitive processing |  |  |  |  |  |
| Cluster # | x | y | z | ALE | Label |
| 1 | 8 | 4 | 12 | 0.047266 | Right Cerebrum.Sub-lobar. Caudate. |
| 1 | 14 | 6 | 0 | 0.042621 | Right Cerebrum.Sub-lobar. Lateral Globus Pallidus |
| 1 | -10 | -2 | 14 | 0.040866 | Left Cerebrum.Sub-lobar. Caudate. |
| 1 | -8 | 10 | -2 | 0.040428 | Left Cerebrum.Sub-lobar. Caudate. |
| 1 | -10 | 2 | 12 | 0.037655 | Left Cerebrum.Sub-lobar. Caudate |
| 1 | 22 | -6 | -8 | 0.028619 | Right Cerebrum.Sub-lobar. Lateral Globus Pallidus |
| 1 | 14 | -4 | 22 | 0.028066 | Right Cerebrum.Sub-lobar. Caudate. |
| 1 | 24 | -8 | 2 | 0.024022 | Right Cerebrum.Sub-lobar. Lateral Globus Pallidus |
| 1 | 30 | -4 | -2 | 0.022584 | Right Cerebrum.Sub-lobar. Putamen |
| 2 | -6 | 38 | 16 | 0.031412 | Left Cerebrum. Limbic Lobe. Anterior Cingulate. |
| 2 | 6 | 54 | 12 | 0.030731 | Right Cerebrum. Frontal Lobe. Medial Frontal Gyrus. |
| 2 | -8 | 44 | 8 | 0.030226 | Left Cerebrum. Limbic Lobe. Anterior Cingulate. |
| 2 | 2 | 50 | 24 | 0.03013 | Right Cerebrum. Frontal Lobe. Medial Frontal Gyrus |
| 2 | -6 | 40 | 2 | 0.028048 | Left Cerebrum. Limbic Lobe. Anterior Cingulate. |
| 2 | 18 | 48 | 22 | 0.027496 | Right Cerebrum. Frontal Lobe. Superior Frontal Gyrus. |
| 2 | -2 | 52 | 6 | 0.023568 | Left Cerebrum. Frontal Lobe. Medial Frontal Gyrus. |
| 2 | -6 | 28 | 28 | 0.02356 | Left Cerebrum. Frontal Lobe. Medial Frontal Gyrus. |
| 2 | -8 | 28 | 24 | 0.022809 | Left Cerebrum. Limbic Lobe. Anterior Cingulate. |
| 3 | 0 | 4 | 58 | 0.040162 | Left Cerebrum. Frontal Lobe. Superior Frontal Gyrus. |
| 3 | 0 | 8 | 44 | 0.036585 | Left Cerebrum. Frontal Lobe. Medial Frontal Gyrus. |
| 3 | 2 | 12 | 38 | 0.030312 | Right Cerebrum. Limbic Lobe. Cingulate Gyrus. |
| 3 | 3 | 2.5 | 61 | 0.023568 | Right Cerebrum.Frontal Lobe.Medial Frontal Gyrus. |
| 3 | **-2** | **8** | **56** | 0.024894798 | Left Cerebrum.Frontal Lobe.Superior Frontal Gyrus. |
| 4 | 10 | 26 | 34 | 0.023338918 | Right Cerebrum.Frontal Lobe.Medial Frontal Gyrus. |
| 4 | **14** | **32** | **30** | 0.022682803 | Right Cerebrum.Limbic Lobe.Cingulate Gyrus. |
| 5 | -15 | -34 | 30 | 0.02593104 | Left Cerebrum.Limbic Lobe.Cingulate Gyrus |
| 5 | **0** | **-42** | **32** | 0.027537983 | Left Cerebrum.Limbic Lobe.Cingulate Gyrus |
| 6 | -10 | 43 | 16 | 0.024498507 | Left Cerebrum.Frontal Lobe.Medial Frontal Gyrus |
| 6 | **-10** | **36** | **16** | 0.015899584 | Left Cerebrum.Limbic Lobe.Anterior Cingulate |
| 6 | -10 | 32 | 20 | 0.01901856 | Left Cerebrum.Limbic Lobe.Anterior Cingulate |
| Conjunction |  |  |  |  |  |
| Cluster # | x | y | z |  | Label |
| 1 | 2 | 8 | 53 |  | Right Cerebrum. Supp_Motor_Area_R |
| 1 | -4 | 16 | 52 |  | Left Cerebrum. Supp_Motor_Area_L |
| 2 | -4 | 12 | 44 |  | Left Cerebrum. Limbic Lobe. Cingulate Gyrus. |
| 2 | 4 | 26 | 34 |  | Right Cerebrum. Limbic Lobe. Cingulate Gyrus |
| 3 | -4 | 28 | 30 |  | Left Cerebrum. Limbic Lobe. Anterior Cingulate |
| 4 | -14 | -4 | 19 |  | Left Cerebrum. Sub-lobar. Caudate. |
| 5 | -14 | -4 | 14 |  | Left Cerebrum. Thalamus_L |
| 6 | -8 | 50 | 8 |  | Left Cerebrum. Frontal_Sup_Medial_L |
| 7 | -80 | 80 | -4 |  | Left Cerebrum. Frontal Lobe. Frontal_Mid_L |

Table 4 Detail peak cluster information for controls in reward and cognitive processing

| Cluster # | x | y | z | ALE | Label |
| --- | --- | --- | --- | --- | --- |
| Reward processing: Controls | | | | | |
| 1 | 18 | 6 | -2 | 0.024895 | Right Cerebrum.Putamen |
| 1 | 8 | 4 | 12 | 0.023339 | Right Cerebrum.Caudate |
| 1 | 14 | -2 | 22 | 0.022683 | Right Cerebrum. Caudate |
| 1 | 12 | 16 | 8 | 0.022403 | Right Cerebrum.Caudate |
| 2 | 0 | 4 | 60 | 0.025931 | Left Cerebrum.Medial Frontal Gyrus |
| 3 | 6 | 54 | 12 | 0.027538 | Right Cerebrum. Medial Frontal Gyrus |
| 4 | -8 | 8 | -2 | 0.024499 | Left Cerebrum. Caudate |
| 4 | -4 | 8 | 6 | 0.0159 | Left Cerebrum.Caudate |
| 5 | -2 | -38 | 30 | 0.019019 | Left Cerebrum.Cingulate Gyrus |
| Cognitive processing: Controls | | | | | |
| 1 | -2 | 24 | 28 | 0.048548 | Left Cerebrum Cingulate Gyrus |
| 1 | -2 | 38 | 24 | 0.037906 | Left Cerebrum Anterior Cingulate |
| 1 | -2 | 14 | 38 | 0.034712 | Left Cerebrum. Cingulate Gyrus |
| 1 | 2 | 12 | 58 | 0.032725 | Right Cerebrum Superior Frontal Gyrus |
| 1 | -2 | 24 | 38 | 0.031257 | Left Cerebrum.Frontal Lobe.Cingulate Gyrus |
| 1 | -4 | 18 | 46 | 0.03015 | Left Cerebrum. Medial Frontal Gyrus |
| 1 | 8 | 20 | 52 | 0.025259 | Right Cerebrum.Superior Frontal Gyrus |
| 1 | -6 | 42 | 16 | 0.023701 | Left Cerebrum.Medial Frontal Gyrus |
| 2 | -10 | -2 | 10 | 0.036276 | Left Cerebrum.Sub-lobar.Thalamus |
| 2 | -12 | 10 | -6 | 0.033095 | Left Cerebrum.Putamen |
| 2 | -8 | 6 | 0 | 0.029197 | Left Cerebrum.Caudate |
| 3 | -52 | 8 | 34 | 0.054404 | Left Cerebrum.Middle Frontal Gyrus. |
| 4 | 24 | 8 | -6 | 0.034044 | Right Cerebrum.Putamen |
| 4 | 16 | 6 | -2 | 0.029754 | Right Cerebrum.Putamen |
| 4 | 10 | 10 | 2 | 0.02884 | Right Cerebrum.Caudate |
| 4 | 12 | 6 | 8 | 0.028179 | Right Cerebrum.Sub-lobar.Caudate |
| 5 | 34 | 20 | 2 | 0.040059 | Right Cerebrum.Sub-lobar.Insula |
| 6 | -32 | 22 | 0 | 0.032737 | Left Cerebrum.Sub-lobar.Insula |
