# Supplementary figures and images for: Common and separable neural alterations in substance use disorders: A coordinate‐based meta‐analyses of functional neuroimaging studies in humans
Source: Hum Brain Mapp. 2020 Sep 10;41(16):4459–77. doi: 10.1002/hbm.25085 (PMC7555084; doi:10.1002/hbm.25085)

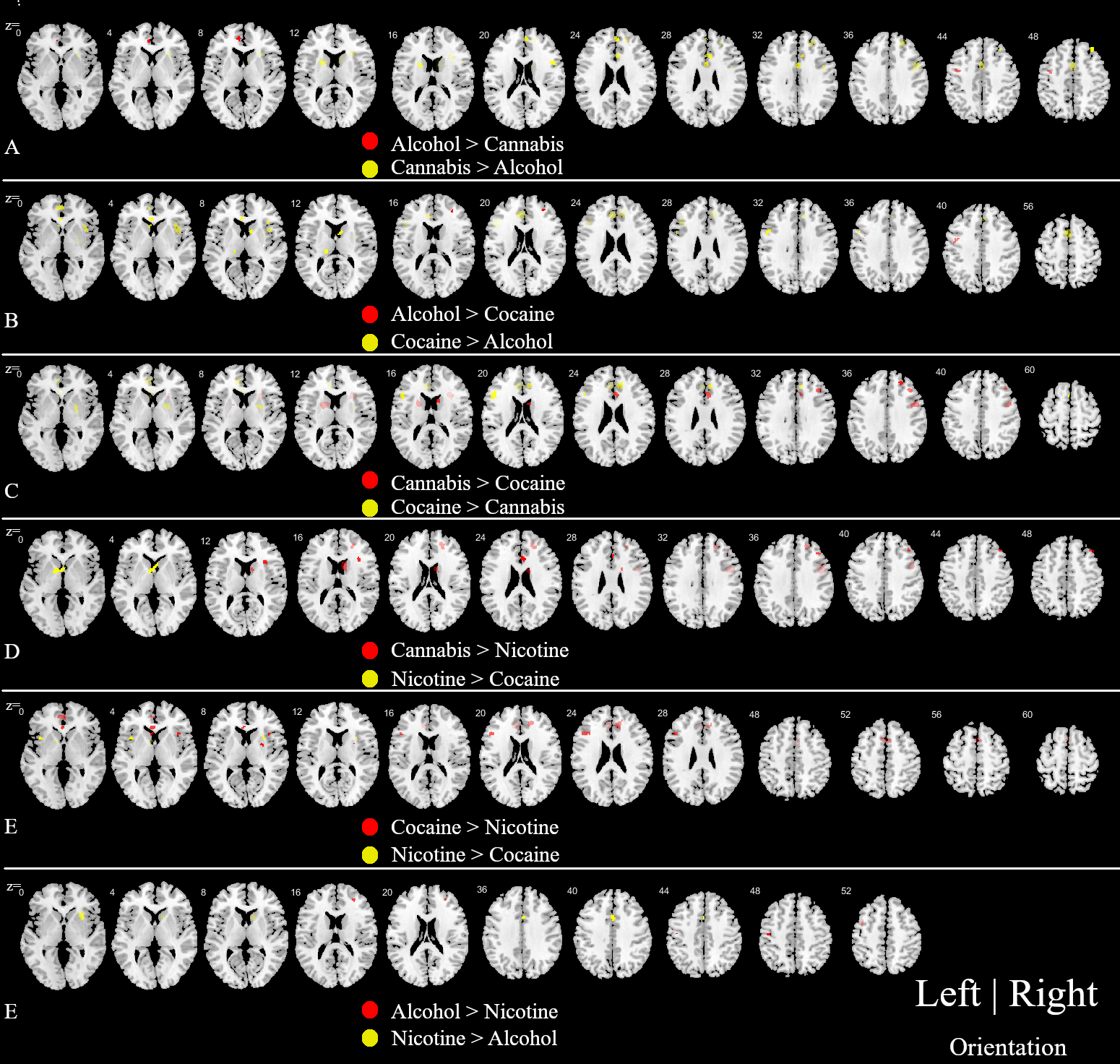

Supplement: Supplementary file 2 — Figure S1 Subtraction analysis between pairs of studies.“>” the symbol indicates where ALE peaks are greater in one study compared to the other. Cluster forming (p < .001) and cluster‐level threshold (p < .05) [file HBM-41-4459-s001.tif]

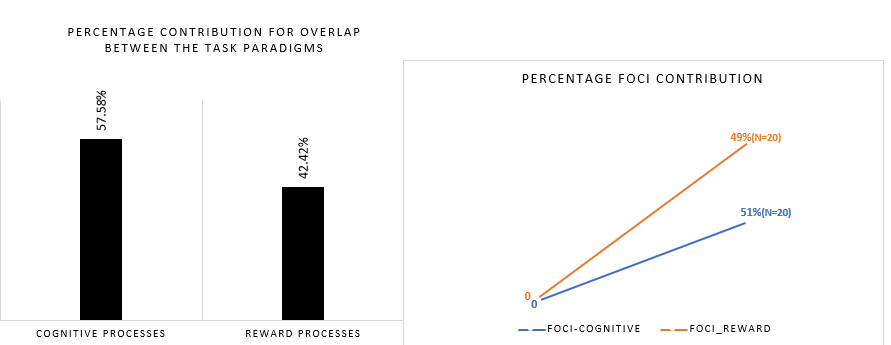

Supplement: Supplementary file 3 — Figure S2 Contributions to the conjunction between the two category of task paradigm, A) the Percentage distribution of each task paradigm to the conjunctions, B) the percentage distribution of foci to the conjunction. N; the number of foci per paradigm [file HBM-41-4459-s003.tif]

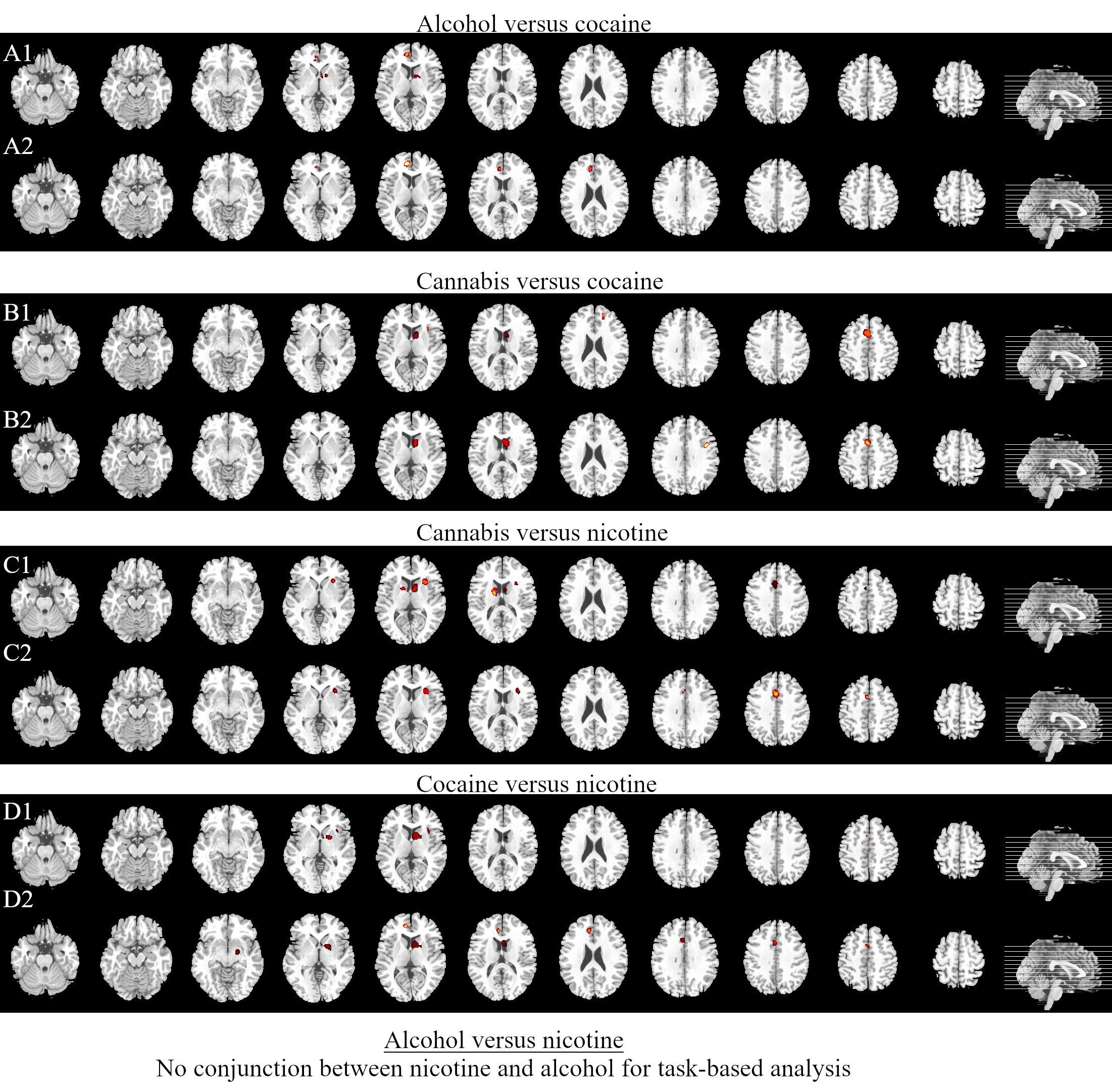

Supplement: Supplementary file 4 — Figure S3 Comparing conjunction based on studies and tasks. Each row Figure label shows conjunction based on studies (see Figure 4 in the main paper) and row label 2 shows conjunction based on task respectively [file HBM-41-4459-s004.tif]
